# Vesicle-associated exudates from *Alteromonas* enhance growth and survival of *Prochlorococcus* in batch culture

**DOI:** 10.1128/aem.00798-26

**Published:** 2026-08-04

**Authors:** Zhiying Lu, Sydney Plummer, James Kizziah, Steven J. Biller, J. Jeffrey Morris

**Affiliations:** 1 Department of Biology, University of Alabama at Birmingham 164521, Birmingham, Alabama, USA; 2 Department of Microbiology, University of Alabama at Birmingham 318277, Birmingham, Alabama, USA; 3 Department of Biological Sciences, Wellesley College 8456, Wellesley, Massachusetts, USA; University of Illinois Urbana-Champaign, Urbana, Illinois, USA

**Keywords:** black queen hypothesis, facilitation, extracellular vesicles

## Abstract

The cyanobacterium *Prochlorococcus* MIT9312 requires helping functions from co-existing organisms for survival under stressful conditions. Here, we show that the helper strain *Alteromonas macleodii* EZ55 also facilitates greater exploitation of medium resources by *Prochlorococcus*. The presence of *Alteromonas* allowed *Prochlorococcus* to grow to greater cell densities, delay growth cessation, and avoid cell death relative to axenic cultures, phenotypes potentially associated with *Alteromonas*-driven degradation of autotoxic substances made by *Prochlorococcus*. We found that heat-labile high-molecular-weight *Alteromonas* exudates mediated the beneficial impacts on *Prochlorococcus*, although it is unclear which specific exudate-associated activities were responsible. Both the composition and activity of the secreted material changed after 500 generations of experimental evolution, suggesting genetic control. Some exudates were located within extracellular vesicles, which were capable of physically associating with *Prochlorococcus* cells and were responsible for at least some of the growth enhancement effects. Many of the functionalities observed in *Alteromonas* exudates (e.g., increasing phosphate availability, hydrogen peroxide degradation, and siderophore capabilities) are consistent with leaky Black Queen processes (i.e., services provided by one organism that benefit the entire community) and favor the evolution of interdependencies in microbial communities. We discuss the potential ramifications of such processes being packaged into vesicles as opposed to freely diffusing through the extracellular milieu.

**IMPORTANCE:**

Heterotrophic bacteria can facilitate improved growth for numerous phytoplankton, including the highly abundant cyanobacterium *Prochlorococcus*. Here, we show that for the “helper” interaction between *Alteromonas* and *Prochlorococcus*, this facilitation is relevant across all phases of batch culture growth and is mediated by secreted products, including proteins and membrane vesicles. We explore the composition of these exudates and show evidence that they are altered during evolutionary adaptation in a changed environment.

## INTRODUCTION

In the temperate and tropical oligotrophic ocean, some free-living marine microbes have undergone substantial genome reduction, forcing them to depend on other microbes to help them by providing the products of these lost genes (1). The Black Queen (BQ) Hypothesis is an evolutionary explanation for how genome reduction may be advantageous for free-living microorganisms in well-mixed environments and may lead members of communities to rely on each other in the absence of kin selection or reciprocation. The BQ Hypothesis states that any biological function that yields products or services that cannot be entirely selfishly retained by the organism performing the function (i.e., the function is *leaky*) provides an opportunity for some lineages in the community to live more efficiently by losing such a function’s genes in order to conserve limited resources (2, 3). Examples of genes encoding leaky functions are those that remove toxins from the extracellular environment (e.g., catalase [4, 5]), produce secreted products (e.g., iron acquisition siderophores [6]), or degrade catabolically desirable polymers in the periplasm or at the cell surface (e.g., alkaline phosphatase, glycolytic enzymes, or peptidases [7–9]).

The “helper” dynamic between *Prochlorococcus* and *Alteromonas* strains in co-culture experiments is both ecologically relevant and a model for studying BQ evolution (10–14). *Prochlorococcus* is the numerically dominant phytoplankton group throughout the temperate and tropical open ocean gyres, where its small size and efficient metabolism allow it to outcompete larger, more complex phototrophs under oligotrophic conditions (15). The high-light ecotypes of *Prochlorococcus* have streamlined genomes lacking enzymes that remove reactive oxygen species (16), leaving them dependent on bacteria like *Alteromonas*, which are found throughout tropical and temperate oceans (17), to mitigate oxidative stress generated by a variety of stressful conditions (18–20). *Alteromonas,* in turn, requires *Prochlorococcus* photosynthate to grow in carbon-free culture media. A previous study suggested that removal of hydrogen peroxide was sufficient to explain the growth enhancement effect of co-culturing *Prochlorococcus* with *Alteromonas*, particularly during early growth stages (11). However, we were interested to know if *Alteromonas* or *Prochlorococcus* modified the culture environment in other ways that might contribute to, or detract from, *Prochlorococcus* growth. Here, we showed that *Prochlorococcus* cultures also benefited from *Alteromonas*’ presence at later stages of batch culture, suggesting the existence of BQ dependencies in addition to ROS protection. We also showed that most of this facilitation was associated with biologically active extracellular products, some of which were contained in extracellular membrane vesicles (EVs) that could associate with *Prochlorococcus* cells. These data suggest EVs may serve as a packaging mechanism for preventing the inefficient dilution of BQ functions in dilute oligotrophic planktonic communities.

## MATERIALS AND METHODS

### Strains and culture conditions

In order to investigate the impact of evolution on the helper interaction between *Alteromonas* and *Prochlorococcus*, all strains used in this study were taken from those used for a long-term phytoplankton evolution (LTPE) experiment (21), in which substantial genomic evolution of both organisms occurred. *Prochlorococcus* strains were streptomycin-resistant derivatives of the high light-adapted strain MIT9312 obtained as described previously (10, 11), either before (“ancestor”) or after 500 generations of evolution (“evolved”) at either 400 ppm or 800 ppm pCO_2_ conditions (i.e., modern-day or projected year 2100 conditions [22]). *Alteromonas* strains were derivatives of strain EZ55, originally isolated from a *Prochlorococcus* MIT9215 culture (10). As with our *Prochlorococcus* strains, we used both ancestral and evolved varieties of EZ55 co-evolved with *Prochlorococcus* at the two pCO_2_ treatments and subsequently isolated into pure culture. *Prochlorococcus* cultures were revived from cultures cryopreserved with 7.5% DMSO in liquid nitrogen vapor, and *Alteromonas* cultures were revived from cultures preserved with 20% glycerol stored at −80°C. Prior to use in experiments, all *Prochlorococcus* cultures were grown in co-culture with *Alteromonas* EZ55 helpers (10) and were acclimated to culture conditions for at least four generations prior to data collection.

*Alteromonas* cultures were grown in YTSS medium (23), and *Prochlorococcus* cultures were grown in Pro99 medium (24, 25) or PEv medium (21), both made in an artificial seawater base (ASW) (21). Prior to addition to co-cultures, *Alteromonas* strains were pelleted at 2,000 × *g* for 2 min and washed twice in sterile ASW, and then added to cultures at approximately 10^6^ cells mL^−1^. *Alteromonas* was grown at 30°C, with shaking at 120 rpm. Unless otherwise noted, *Prochlorococcus* and co-cultures were grown in static 13 mL conical bottom acid-washed glass tubes under approximately 75 μmol photons m^−2^ s^−1^ cool white light in a Percival incubator set to 23°C. When medium additions were employed, all solutions were filter sterilized with a 0.2 μm filter. Cell densities of *Prochlorococcus* cultures to standardize inoculations between experiments were determined using a Guava HT1 flow cytometer (Luminex Corporation, Austin, TX) by the distinctive signature of these cells on plots of forward light scatter vs red fluorescence (Fig. S1A). Day-to-day culture growth was tracked using the *in vivo* chlorophyll *a* module for the Trilogy fluorometer (Turner Designs, San Jose, CA) with a custom 3D-printed adapter designed for conical bottom tubes. Fluorometer measurements and cell counts were linearly related across the range of cells examined in this study (Pearson correlation coefficient 0.835, *P* = 1.38 × 10^−6^; Fig. S1B).

### Growth tests in conditioned media

We conducted tests using three types of conditioned media: *Prochlorococcus* (Pro CM), *Alteromonas* (EZ55 CM), and *Prochlorococcus* subsequently treated with *Alteromonas* (Pro CM + EZ55). For Pro CM, we produced axenic *Prochlorococcus* by adding streptomycin to a final concentration of 100 μg mL^−1^ to low-density (<10^6^ cells mL^−1^) *Prochlorococcus* cultures as described previously (11). After 48 h exposure to the antibiotic, we confirmed that no *Alteromonas* EZ55 cells survived by transferring 1 mL into sterile YTSS medium and checking for growth by visual inspection of optical density after 24 h. A 0.5 mL aliquot of this axenic *Prochlorococcus* culture was transferred to 12 mL fresh Pro99 medium and cultivated for 11 days (past the end of exponential growth), after which the cells were removed by filtering the medium using a sterile 0.2 μm Millex-GV PVDF syringe filter (Millipore Sigma, Burlington, MA, USA). EZ55 CM and Pro CM + EZ55 were produced by inoculating washed *Alteromonas* EZ55 cells from YTSS medium at approximately 10^6^ CFU mL^−1^ to sterile Pro99 (EZ55 CM) or to a sub-sample of the Pro CM described above (Pro CM + EZ55). As with Pro CM, these cultures were cultivated for 11 days and were then filtered to remove the cells; previous work showed that *Alteromonas* cell densities were stable in ASW without added C over this time frame (12). Sterility of the filtrates was confirmed by the same method used to confirm axenicity as described above. To initiate experiments, freshly axenic *Prochlorococcus* (produced as described above) was transferred to triplicate 12 mL tubes of each of the three conditioned media, and growth was measured by chl-*a* fluorescence every other day. For these experiments, ancestral *Prochlorococcus* was paired with CM from ancestral *Alteromonas,* and evolved *Prochlorococcus* was paired with CM from *Alteromonas* isolates obtained from the same evolved *Prochlorococcus* culture.

### Concentration of *Alteromonas* exudates and purification of EVs

EZ55 was grown in Pro99 media supplemented with 0.1% glucose to sustain growth in the absence of *Prochlorococcus* exudates. We scaled cultures up progressively from 12 mL to 2 L. The 2 L culture was grown in a vented bottle with an outlet connected to a filter with 0.22 μm pore size. After removing most of the cells by centrifugation, we produced size-fractionated, concentrated exudates using tangential flow filtration using Sartorius Vivaflow 200 cassettes. The 2 L culture supernatant was passed first through a 0.22 μm cassette using a Masterflex L/S peristaltic pump (Cole-Parmer) to remove bacterial cells and then through a 50 kDa module and a 5 kDa module in succession to produce >50 kDa and <50 kDa fractions that were each concentrated approximately 100-fold. These fraction sizes were chosen to separate large soluble protein complexes and EVs (>50 kDa), small peptides and oligonucleotides such as signaling molecules (<50 kDa but >5 kDa), and other small metabolites (<5 kDa) to determine the relative impact of molecules in these size ranges on the observed culture responses. A portion of the >50 kDa fraction was placed in boiling water for 5 min to denature proteins. When these concentrated extracellular products were added to culture media for growth experiments, they were diluted 100-fold, returning them to approximately their original concentration prior to filtration.

To further purify EVs, the >50 kDa fraction described above was passed through a 0.22 µm syringe filter and then processed by ultracentrifugation (Beckman Coulter Optima X Series) at 38,000 rpm (106,255 × *g*) for 3 h at 4°C using a Ti-70 rotor (Beckman Coulter). The supernatant was decanted, and the pellet was washed with sterile Pro99 + 0.1% glucose; this step was repeated for two washes per sample. We then adapted methods from a previous study (26) to perform an ultracentrifugation density gradient using CsCl. Briefly, the sample layer was gravimetrically adjusted to 1.45 g/mL^−1^ with a 1.9 g/mL^−1^ CsCl solution. Then, a density gradient was prepared by carefully overlaying density layers in the following order: 1.6 g CsCl/mL^−1^, 1.45 g/mL^−1^ sample layer, and 1.2 g CsCl/mL^−1^. All CsCl solutions were prepared in SM buffer (100 mM NaCl, 8 mM MgSO_4_, 50 mM Tris-HCl, pH = 7.5). The density gradient was ultracentrifuged at 30,000 rpm (154,000 × *g*) and 4°C for 72 h using a SW Ti 41 rotor (Beckman Coulter). Fractions were carefully removed with a pipette, and weights were recorded. Finally, CsCl was removed by performing a buffer exchange with TE buffer (10 mM Tris–HCl, 1 mM disodium EDTA, pH 7.5) using centrifugal filter units (Amicon Ultra Filter Device UFC9010; 10 kDa MWCO) and spinning at 4,000 × *g* for 30 min at 4°C with a swinging bucket rotor and benchtop centrifuge. Concentrated and purified EVs were collected from the least dense fraction and stored in aliquots at −80°C prior to use in experiments. Where indicated, EVs were added to cultures to a final concentration of 10^8^ EVs/mL^−1^, with the concentration determined as described below.

### Proteomics

The >50 kDa fraction described above was further concentrated using a 30 kDa centrifugal filter (MilliporeSigma Amicon Ultra-15, Darmstadt, Germany) to ~1.5 mL by centrifugation at 7,000 × *g*. Then, 13.5 mL sterile Milli-Q water was added to the filtrate and was concentrated to ~1.5 mL again. The above wash step was repeated, and the final ~1.5 mL sample was transferred to a sterile 2 mL tube for storage at 4°C. We also isolated proteins from whole EZ55 cells from the same cultures used to produce the >50 kDa fraction using a Bacterial Cell Lysis kit (GoldBio). The total protein concentration for each sample was measured using a DC Protein Assay Kit (Bio-Rad, Hercules, CA, USA). The samples were then diluted with 4× Laemmli Sample Buffer (Bio-Rad, Hercules, CA, USA) containing 2-mercaptoethanol (Bio-Rad, Hercules, CA, USA) at the rate of three parts sample to one part buffer. The diluted sample was heated at 95°C for 5 min, and 20 μL was loaded onto a 4%–20% Mini-PROTEAN TGX precast polyacrylamide gel (Bio-Rad, Hercules, CA, USA). Gel electrophoresis was performed in a vertical direction in a Mini-PROTEAN Tetra cell (Bio-Rad, Hercules, CA, USA) at ~200 V for 20–40 min until the blue band in the marker line reached the bottom of the gel. After electrophoresis was complete, the gel was gently removed from the cassette and was rinsed in a shallow staining tray with Milli-Q water. The rinsed gel was soaked in fixing solution (40% ethanol and 10% acetic acid) for 15 min with gentle agitation, rinsed with Milli-Q water again, and stained with colloidal Coomassie blue for 14 h with gentle agitation at room temperature. The stained gel was destained in three changes of Milli-Q water over 3 h with gentle agitation.

For protein identification, the portion of the destained gel containing target bands of interest was cut into eight slices with equal length (Fig. S2), and each slice was digested following the In-Gel Digestion Protocol described by a previous study (27). Each digest was analyzed as previously described (28). An aliquot (5 μL) of each digest was loaded onto a Nano cHiPLC 200 μm ID × 0.5 mm ChromXP C_18_ -CL 3-μm 120-Å reverse-phase trap cartridge (Eksigent, Dublin, CA) at 2 μL/min using an Eksigent 415 LC pump and autosampler. After the cartridge was washed for 10 min with 0.1% formic acid in ddH_2_O, the bound peptides were flushed onto a Nano cHiPLC 200-μm ID × 15-cm ChromXP C -CL 3-μm 120-Å reverse-phase column (Eksigent) with a 100-min linear (5 to 50%) acetonitrile gradient in 0.1% formic acid at 1,000 nL/min. The column was then washed with 90% acetonitrile + 0.1% formic acid for 5 min and re-equilibrated with 5% acetonitrile + 0.1% formic acid for 15 min. A Sciex 5600 Triple-TOF mass spectrometer (Sciex, Toronto, Canada) was used to analyze the protein digest. The IonSpray voltage was 2,300 V, and the declustering potential was 80 V. Ion spray and curtain gases were set at 10 and 25 lb/in^2^, respectively. The interface heater temperature was 120°C. Eluted peptides were subjected to a time-of-flight survey scan from m/z 400 to 1,250 to determine the top 20 most intense ions for tandem mass spectrometry (MS/MS) analysis. Product ion time-of-flight scans (50 ms) were carried out to obtain the MS/MS spectra of the selected parent ions over the range from m/z 400 to 1,000. The spectra were centroided and deisotoped by Analyst software (v1.7 TF; Sciex). A β-galactosidase trypsin digest was used to establish and confirm the mass accuracy of the mass spectrometer.

The MS/MS data were processed to provide protein identifications using an in-house Protein Pilot 4.5 search engine (Sciex) using the NCBI non-redundant protein database and a trypsin digestion parameter and carbamidomethylation for alkylated cysteines as a fixed modification. Proteins of significance were accepted based on the criteria of having at least two peptides detected with a confidence score of >95% using the Paradigm method embedded in the Protein Pilot software. Complete amino acid sequences of predicted proteins were downloaded using the Bio.Entrez package from BioPython (29). Subcellular localization of proteins was predicted using PSORTb v 3.0 (30). KEGG orthology group codes were obtained for proteins using BlastKOALA (31) and were binned into pathways using KEGGREST (32) in R (33). Estimated molecular weights for EZ55 proteins were calculated using the CusaBio molecular weight calculator (https://www.cusabio.com/m-299.html). Data were statistically analyzed and visualized within R.

### Cryo-electron microscopy (cryo-EM)

Samples of the concentrated and desalted >50 kDa fractions were kept in a cool box during transport to the UAB Cryo-EM facility, where they were immediately cryogenically frozen in preparation for microscopy as previously described (12). Briefly, 3 μL of each sample was applied to glow-discharged Quantifoil R2/2 200 mesh nickel grids (Electron Microscopy Sciences, Hatfield, PA, USA) and vitrified in liquid ethane using an FEI Vitrobot Mark IV (Thermo Fisher Scientific). The grids were observed on an FEI Tecnai F20 electron microscope (Thermo Fisher Scientific) operated at 200 kV with a typical magnification of ~34,500× and 1–3 μm defocus. In total, 111 images were collected under low-dose conditions in SerialEM using a Gatan K3 direct detector. For single particle reconstruction, micrographs were imported to RELION-3 (34), and contrast transfer functions were estimated using Gctf (35). Particles were picked with Topaz (36) using a default model (resnet8_u64). A subset of highly symmetric particles was isolated via iterative 2D classification and then separated for 3D reconstruction. Matches to modeled structures were searched in the Protein Data Bank, and these structures were then fit to the reconstruction using ChimeraX (37).

The diameters of vesicles from CryoEM micrographs were measured manually using the Straight Line Tool in ImageJ (v1.54p) (38). The scale was set to 1.474 pixels Å^−1^, which reflects the pixel size at which the images were measured. A Gaussian Blur with sigma = 1 was applied to reduce noise. For some dark and noisy images (<10 images), the contrast was also enhanced by saturating pixels by 0.3%. Diameter in Å was then converted to nm.

### Exudate interaction with *Prochlorococcus*

In order to visualize interactions between exudates and *Prochlorococcus* cells, we covalently labeled the >50 kDa concentrates with an amine-reactive Alexa Fluor 488 5-SDP ester dye (Molecular Probes/Thermo Scientific) (39). First, 100 μL of each sample was concentrated with 100 kDa centrifugal ultrafilters (Pall) at 14,000 rpm for 15 min and then resuspended in 0.1 M sodium bicarbonate buffer (pH 8.3). Then, 2.5 μL of Alexa Fluor 488 5-SDP ester dye was added and incubated for 1 h in the dark at room temperature with gentle mixing. The mixture was washed by centrifugation at 14,000 rpm for 15 min, removal of the supernatant, and resuspension with 1× PBS three times, with a final resuspension in 100 μL ASW. The labeled and washed exudate was added to 100 μL axenic *Prochlorococcus* culture and incubated for 2 h at 28℃, with 120 rpm orbital shaking. Control samples were prepared with *Prochlorococcus* cells only or with *Prochlorococcus* stained with Alexa Fluor 488 5-SDP ester dye but without adding exudate concentrate. Cells were analyzed with a Guava HT1 flow cytometer tracking cells by size (forward scatter), chlorophyll (red) fluorescence, and dye (green) fluorescence. Flow cytometry data were visualized using the online Floreada platform (https://floreada.io). Cells were also examined using a Nikon 80i epifluorescence compound microscope with the GFP and Texas Red filter cubes. Images were collected and processed using Nikon NIS-Elements imaging software.

### Biological activities of exudates

We used a variety of enzymatic tests to evaluate the activity of *Alteromonas* exudates. Specifically, the fluorescent conjugates 4-nitrophenyl-α-D-glucopyranoside, 4-nitrophenyl-β-D-glucopyranoside, and 4-nitrophenyl-N-acetyl-β-D-glucopyranoside were used to measure the activities of the glycolytic enzymes α-glucosidase, β-glucosidase, and N-acetyl-glucosaminidase, respectively (40). Phosphatase activity was measured by following the hydrolysis of fluorescent substrate 4-methylumbelliferyl phosphate (41). Enzyme activity for these experiments was expressed as the rate of fluorescent product accumulation over the first 2–5 h, minus the rate of accumulation in an exudate-free media control. Protease activity was measured after a 30-min incubation based on Sigma’s non-specific protease activity assay using casein as a substrate (42). Siderophore activity was determined after a 30-min incubation using chrome azurol S (43). Both fluorescence and optical density traces for these assays were measured using a Biotek Synergy H1 plate reader (Agilent, Santa Clara, CA). Hydroperoxidase activity was assessed by measuring the elimination of a hydrogen peroxide spike after 2 h, tracking the hydrogen peroxide concentration using acridinium ester chemiluminescence via the injection protocol described previously (11) but modified for use with the Synergy H1. We also attempted to determine the role, if any, of intact EVs in exudate function by sonicating a subsample of the >50 kDa fraction prior to assays. To accomplish this, the concentrates were treated with a Fisher Scientific sonic dismembrator for three cycles of 10 s with a 10 s pause between (100 W, 30% output efficiency). Both methylumbelliferyl and 4-nitrophenyl conjugate substrates are routinely used to measure membrane permeability due to their inability to cross membranes (44, 45); hence, an increase in apparent enzymatic activity post-sonication can be interpreted as an indication of membrane disruption. While sonication may also disrupt protein complexes, this would be expected to reduce, not increase, apparent enzyme activity; hence, we concluded that an increase in activity post-sonication reflected EV disruption.

### Extracellular vesicle concentration and siz*e*

EV size and concentration were assessed using a NanoSight NS300 (Malvern Panalytical) equipped with a 488 nm laser. Samples were diluted as necessary with sterile Pro99 media to record 10 to 100 vesicles frame^−1^ according to the manufacturer’s protocols. The screen gain and camera levels were set to 1 and 13 for all samples, respectively. Samples were injected with a syringe pump at 50 µL s^−1^. A total of 5 videos at 62 frames per second were recorded for each sample. The sample chamber and tubing were flushed with Milli-Q water between samples. Videos were analyzed with the NanoSight NTA version 3.4 software (Malvern Panalytical) with the detection threshold set to 5. Vesicle concentrations in cultures were calculated with the following equation:


$$C_{culture}=\;C_{NS}\times NS\;dilution\;factor\;\times \left(\frac{V_{UC}}{V_{culture}}\right)$$

where *C*_culture_ is the concentration of particles in the cultures, *C*_NS_ is the concentration detected on NanoSight, NS dilution factor is the dilution factor of samples analyzed on NanoSight, *V*_UC_ is the volume of the culture post-ultracentrifugation, and *V*_culture_ is the volume of the culture pre-ultracentrifugation. The diluent particle concentration was subtracted from the sample particle concentration as necessary.

### Statistical analysis

All statistical analyses were performed in R v. 4.4.1. Most analyses used linear models, followed by post hoc extended marginal means testing of pairwise differences between treatment groups using the *emmeans* package (46). Where no significant difference between tested strains existed, “strain” was removed from analyses as a predictor variable to improve statistical power. Assumptions of linear regression were checked for models by Shapiro-Wilk tests and/or histogram plots for the normality of residuals, and plots of residuals vs fitted values for homoscedasticity; where these assumptions were violated, we used the Box-Cox procedure to find an optimal power transformation (47). Statistical differences between lysate and exudate protein localization counts were determined using Fisher’s exact test implemented in R. Nanosight estimates of EV concentrations were compared using one- or two-sample *t*-tests in R using means and standard deviations output from Nanosight software. Correction for multiple tests was performed automatically by *emmeans* using default settings for linear models and was performed manually using the Holm-Bonferroni method (47) for Fisher’s exact test and *t*-tests.

## RESULTS

### Helper bacteria prolong *Prochlorococcus* growth in batch culture

**Fig 1:**
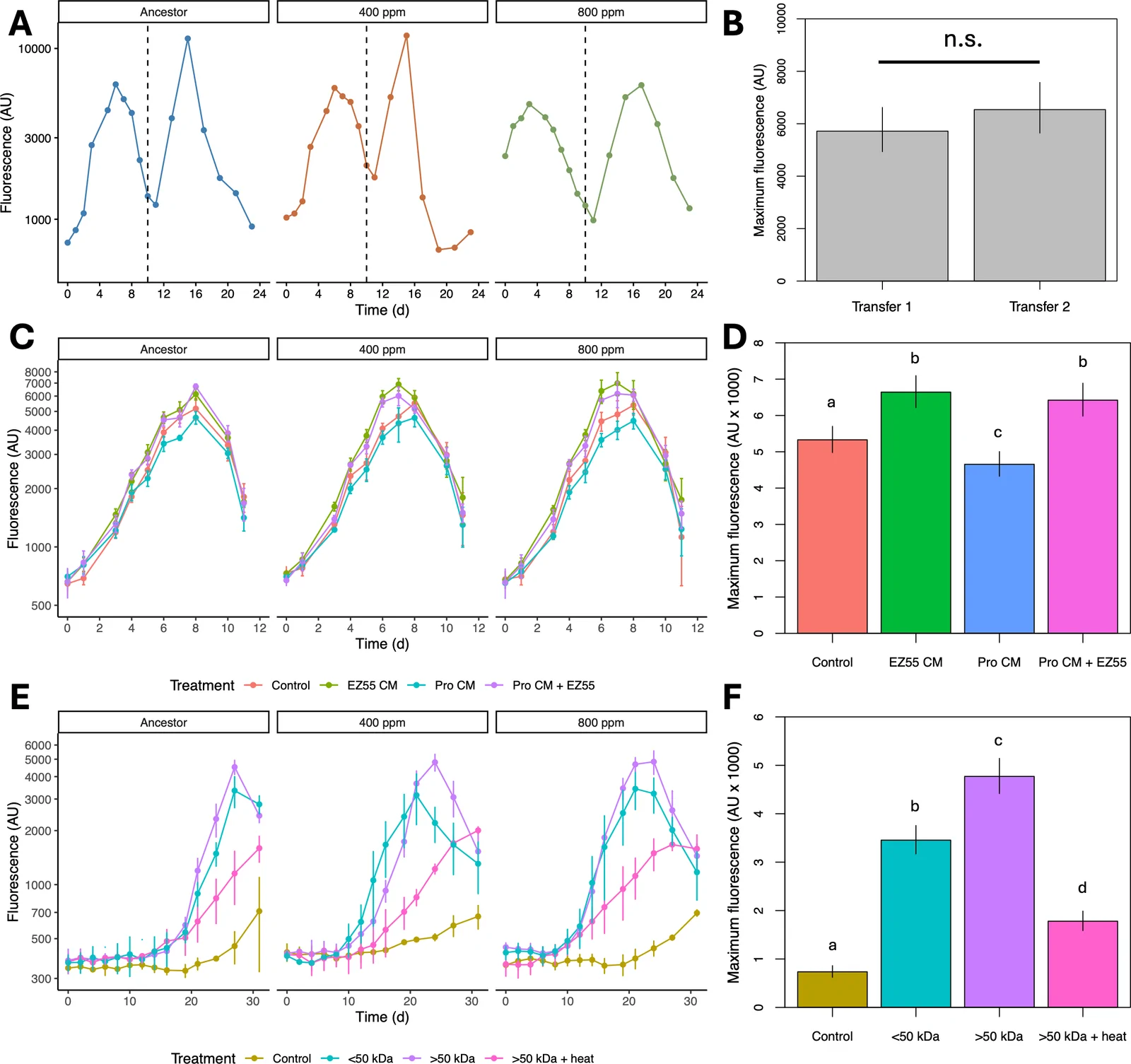
Impact of *Alteromonas* EZ55 exudates on *Prochlorococcus* growth. (**A**) Representative growth curves from *Prochlorococcus*/EZ55 co-cultures grown in Pro99 media and then transferred (at the point marked with a dashed vertical line) into nutrient-free artificial seawater. (**B**) Maximum fluorescence achieved in 14 cultures first grown in Pro99 (Transfer 1) and then diluted into nutrient-free artificial seawater (Transfer 2). n.s., no significant difference. (**C**) Growth curves from axenic *Prochlorococcus* cultures in Pro99 medium that was either unamended (Control) or else conditioned by EZ55 (EZ55 CM) or *Prochlorococcus* either without (Pro CM) or with subsequent treatment with EZ55 cells (Pro CM + EZ55). (**D**) Maximum fluorescence achieved for each treatment in panel C. (**E**) The same *Prochlorococcus* strains grown in Pro99 medium either unamended (Control), with the addition of a concentrated <50 kDa or >50 kDa fraction of the cell-free supernatant from an EZ55 culture, or with the > 50 kDa fraction following heat treatment to denature proteins. (**F**) As panel D, but for the growth curves in panel E. Letters in panels B and D reflect significance groups from a within-strain pairwise Tukey test. Ancestor, 400 ppm, and 800 ppm reflect *Prochlorococcus* and *Alteromonas* strains before or after 500 generations of evolution under altered pCO_2_ conditions. Error bars in B, D, and F represent the 95% confidence intervals from predicted means based on a linear model.

In our laboratory, we routinely maintain *Prochlorococcus* in co-cultures with the helper heterotrophic bacterium *Alteromonas macleodii* EZ55 under low light conditions as seed stocks for experiments. We use two types of media for these cultures: Pro99, a standard medium for *Prochlorococcus* (24, 25), and PEv, a low-nutrient medium designed to more closely reflect *Prochlorococcus*’s native oligotrophic habitat (21). Although PEv has 1/25 the nutrient concentrations of Pro99, we observed that maximum cell densities in these two media types only differed by approximately 5-fold (Fig. S3), suggesting that growth cessation in Pro99 was not induced by nutrient starvation. We confirmed this by diluting post-growth Pro99 cultures 2:1 using either Pro99 (Fig. S4) or nutrient-free artificial seawater (Fig. 1A and Fig. S5); in both cases, culture growth was nearly identical, with statistically indistinguishable maximum cell densities observed between transfers whether additional nutrients were present (Fig. 1B, linear model, *P* > 0.05).

To investigate what other factors might have influenced maximum batch culture cell density, we considered the possibility that the metabolism of dense *Prochlorococcus* cultures may have induced chemical changes in the carbonate system that were toxic to the cells. Like most aquatic phototrophs, *Prochlorococcus* maintains a carbon concentrating mechanism that converts bicarbonate to CO_2_ in the vicinity of Rubisco, consuming protons and raising pH in the process (48). In the late exponential stage cultures, we observed a density-dependent pH increase of approximately 1 unit from 7.9 to 8.9 (Fig. S6), a 10-fold increase in [OH^−^] which would not be substantially alleviated by a 2-fold dilution into fresh media. We also considered the possibility that growth arrest occurred due to carbon limitation, since we grew the cultures in sealed tubes without agitation (25). However, addition of sterile NaHCO_3_ failed to restore growth, arguing against this possibility (Fig. S7).

As we inspected growth curves of *Prochlorococcus* in Pro99 medium, we observed that many cultures experienced pronounced die-offs within a few days of growth cessation (Figs. S4 and S5). Because previous studies revealed that axenic *Prochlorococcus* cultures experienced similar mortality events (12, 21, 49), we investigated the role that the co-cultured helper bacterium *Alteromonas* played in the later stages of batch culture. Axenic cultures ceased growing at significantly lower cell densities than co-cultures with *Alteromonas* (Fig. S8). Moreover, when we diluted post-growth Pro99 co-cultures 2:1 into ASW as above after the onset of mortality, we still observed restored growth, but not in cultures where *Alteromonas* was killed with antibiotics. Rather, these axenic cultures eventually died out completely (Fig. S9). Based on these observations and our experiments with spent supernatants, we concluded that *Prochlorococcus* modified the medium in a way that arrested growth and caused cell death prior to nutrient exhaustion and that *Alteromonas* was able to at least partially ameliorate this condition.

### The helping ability of *Alteromonas* is contained in high-molecular-weight exudates

*Alteromonas* and other microbes protect dilute *Prochlorococcus* cultures from H_2_O_2_ in their environment, necessitated by the relatively low per-cell ability of *Prochlorococcus* to detoxify this compound (11, 18). However, at higher cell concentrations like those studied here, *Prochlorococcus* was able to bring H_2_O_2_ concentrations below the limit of detection in axenic cultures, indistinguishable from co-cultures with *Alteromonas* (11). We therefore hypothesized that some other product of *Alteromonas* was responsible for extending the growth period and allowing higher maximum cell densities of cultured *Prochlorococcus*. To explore this possibility, we inoculated three different *Prochlorococcus* isolates at a high initial cell density of 10^6^ cells mL^−1^ in Pro99 (a minimal seawater medium containing only inorganic salts and trace metals) that had been pre-conditioned by either Pro CM, EZ55 CM, or Pro CM + EZ55 (see Methods) (Fig. 1C). Exponential growth rates generally did not differ between treatments (Fig. S10A), but the maximum cell density (Fig. 1D) and area under the growth curve (Fig. S11A) achieved in EZ55 CM were significantly higher than in fresh Pro99, confirming that bacterial facilitation remained relevant at high cell densities, and not just in dilute cultures as previously shown (10, 11). Interestingly, cultures grown in Pro CM had significantly reduced maximum and integrated fluorescence relative to the fresh Pro99 control, consistent with the accumulation of toxic *Prochlorococcus* exudates in denser cultures. In contrast, maximum and integrated fluorescence in Pro CM + EZ55 were not significantly different from growth in EZ55 CM (Fig. 1D and Fig. S11A), supporting the hypothesis that *Alteromonas* exudates were able to detoxify *Prochlorococcus*’ spent medium. The observed impact of EZ55 CM also indicated that the growth-enhancing factors were both extracellular (as no living *Alteromonas* cells were present in these experiments) and stable for at least a week post-filtration.

We next sought to gain insights into the extracellular *Alteromonas* products contributing to these observations. We size-fractionated and concentrated the contents of all three varieties of EZ55 CM, producing fractions 5–50 kDa (<50 kDa) and >50 kDa. Anticipating that the active components of the higher molecular weight fraction may be proteins or other complex heat-labile molecules, we boiled samples of the >50 kDa fraction to deactivate such compounds. To observe the effect of exudates across the batch culture growth cycle, we then inoculated dilute (~1.5 × 10^4^ cells mL^−1^) axenic *Prochlorococcus* cultures in fresh Pro99 medium into which these fractions were added. Under these conditions, unmodified cultures showed no detectable growth for 25 days, consistent with previous studies showing the dependence of dilute *Prochlorococcus* cultures on bacterial facilitation for survival (10, 11). In contrast, the addition of bacterial exudates dramatically enhanced growth (Fig. 1E). The greatest growth enhancement was delivered by the >50 kDa fraction, which was significantly more effective than the smaller molecular weight fraction in raising maximum cell density (Fig. 1F) and also generally more effective for increasing area under the growth curve (Fig. S11B) and exponential growth rate (Fig. S11B). Heat treatment eliminated most of this helping ability, although the boiled samples still allowed greater growth than unmodified Pro99.

### *Alteromonas* exudates include protein-containing EVs

**Fig 2:**
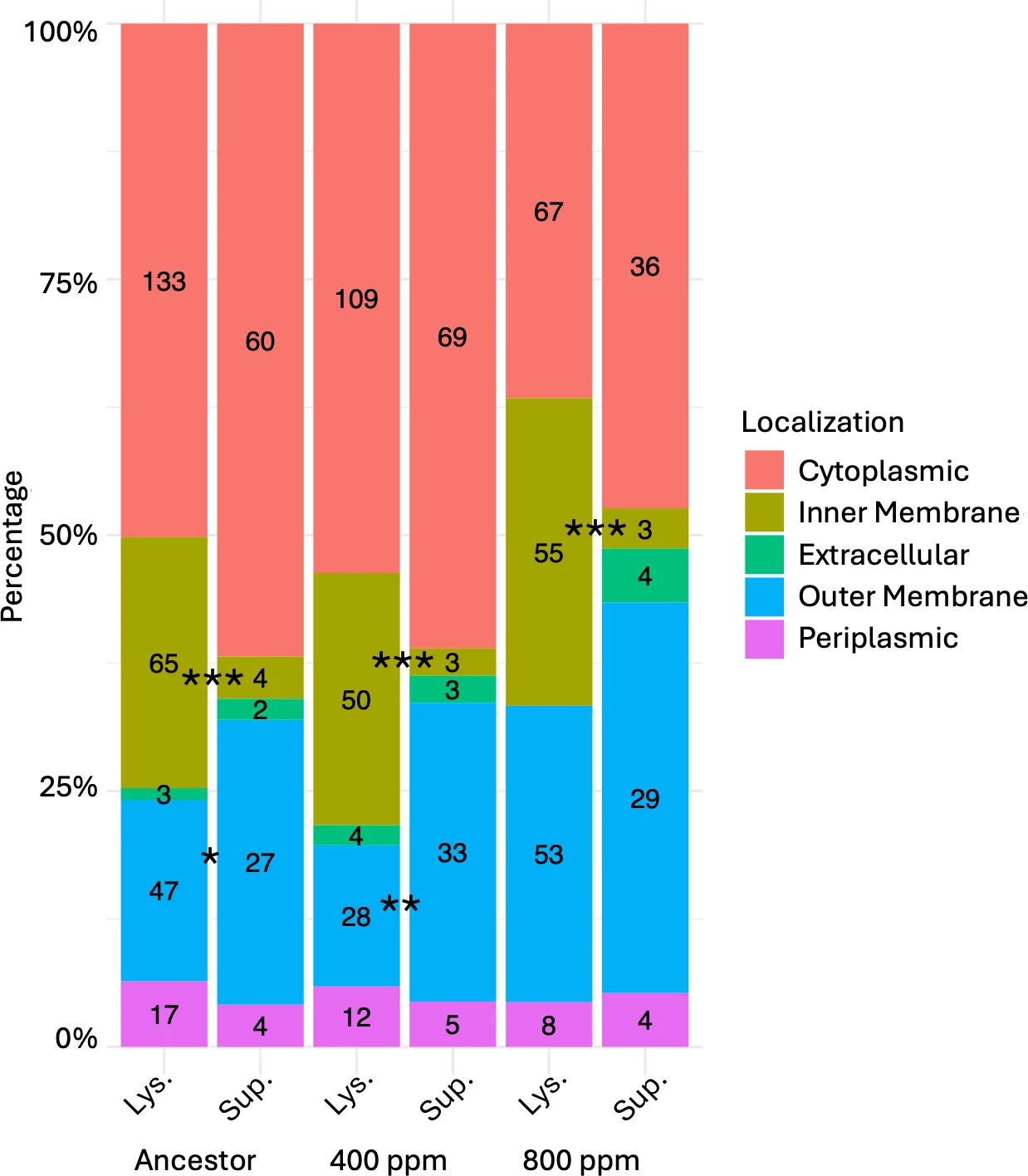
Predicted localization of *Alteromonas* proteins. *Alteromonas* strains were isolated from cultures either before (Ancestor) or after 500 generations of evolution at modern or projected future pCO_2_ conditions (400 ppm and 800 ppm, respectively). Proteins were identified by LC-MS from whole cell lysates (Lys) or from concentrated cell-free supernatants (Sup). Localization of proteins was predicted using psortB. Asterisks indicate the result of Fisher’s exact test comparing counts of the indicated proteins between lysate and supernatant for the given *Alteromonas* strain; *, *P* < 0.05; **, *P* < 0.01; ***, *P* < 0.001.

Based on these results, we investigated the proteins in the >50 kDa supernatant size fraction and compared them to proteins present in crude cell lysates of the *Alteromonas* strains. Overall, 602 peptides corresponding to predicted *Alteromonas* products were detected (Table S1). A total of 68 proteins were detected in the exudates of all three *Alteromonas* isolates, and 144 proteins were detected in the lysates of all isolates (36% and 31% of the total, respectively) (Fig. S12). In contrast, fewer than 10% of proteins were shared between lysates and supernatants (Fig. S13), arguing against the possibility that our supernatant proteins were the result of simple cellular lysis. Surprisingly, fewer than 5% of the detected supernatant proteins were predicted to be extracellular (Fig. 2), indicating that most were not canonically secreted proteins. Unexpectedly, 63% of the proteins found in the >50 kDa supernatant fraction were predicted to be smaller than 50 kDa, including some as small as 5 kDa (Table S1); this value is only slightly lower than the 71% of proteins <50 kDa found in the crude lysates and is consistent with the bands observed on SDS-PAGE gels (Fig. S2). Additionally, the average molecular weight of supernatant proteins was only significantly higher than lysate proteins for one *Alteromonas* strain (Fig. S14) and then only by about 20%. While some of these smaller proteins may exist as multimers in their active configuration or else may be associated with larger molecules such as ribosomes or nucleic acids with which they may have been co-purified, their frequency strongly suggested the presence of an alternative, larger carrier. The functional categories of proteins were diverse and were not significantly different between lysates and supernatants (Fig. S15).

These observations raised the question of why smaller proteins were retained by the 50 kDa molecular weight filter. We realized that these results might occur if the proteins were packaged inside EVs. EVs are small spherical structures, enclosed by a lipid bilayer, which can contain components from the producing cells. Both gram-positive and gram-negative bacteria can produce EVs (50), and they have been observed previously in both *Prochlorococcus* and *Alteromonas* cultures (39, 51, 52). We observed that proteins predicted to localize to the cytoplasmic membrane were significantly depleted in the supernatant fraction of all *Alteromonas* strains and outer membrane proteins were significantly enriched in two of three strains (Fisher’s exact test, *P* < 0.05; Fig. 2), consistent with the presence of EVs primarily comprised of the outer membrane of these gram-negative bacteria and previous analyses of *Alteromonas* EV proteomes (52).

**Fig 3:**
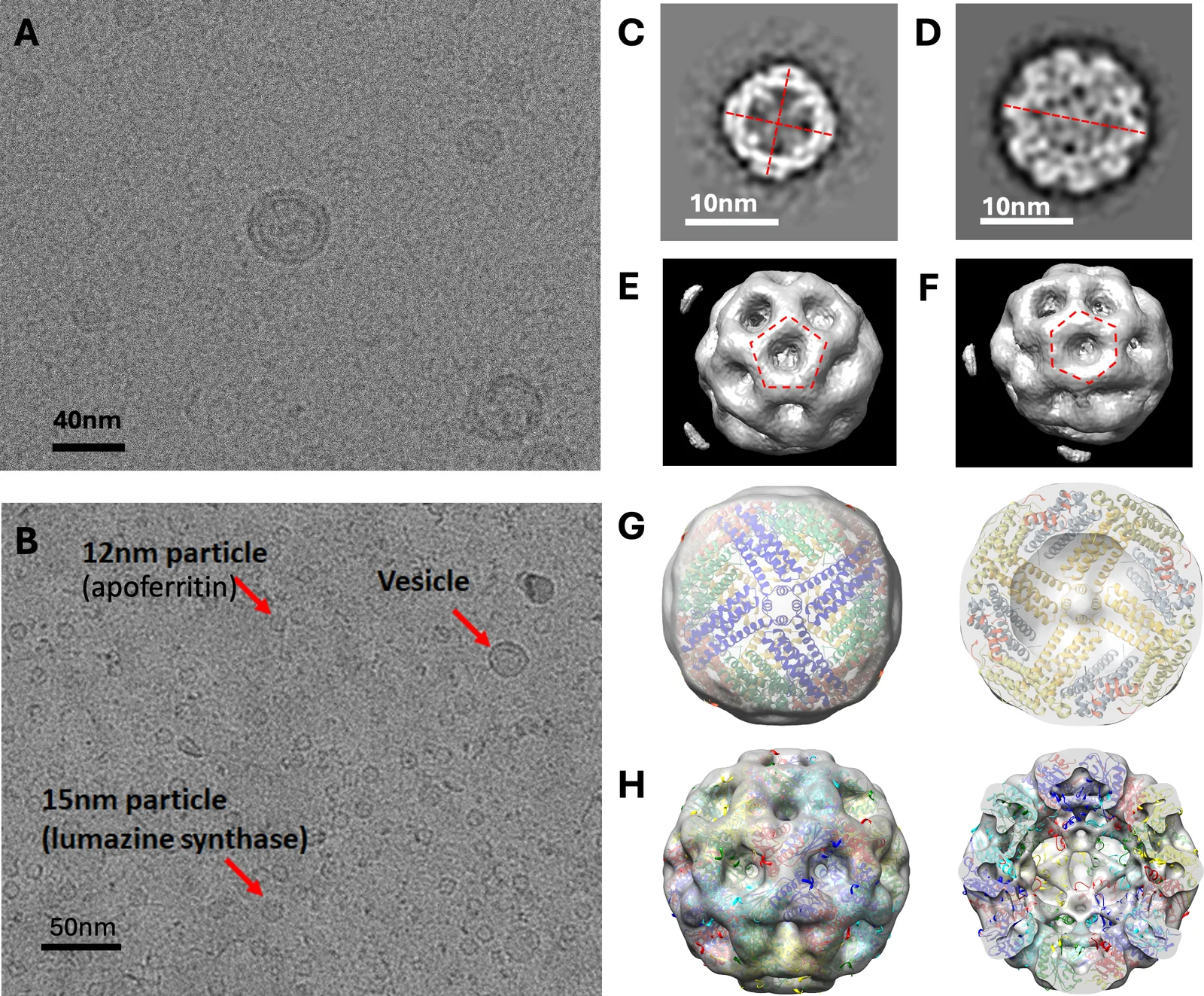
*Alteromonas* exudates contain membrane vesicles and large protein structures. (**A**) Cryo-electron microscopy of concentrated *Alteromonas* exudates revealed abundant bilayer structures resembling membrane vesicles. (**B**) In addition to membrane vesicles, cryo-EM also revealed large proteins and protein complexes in the extracellular milieu. (**C** and **D**) 2D class average images of 12 and 15 nm particles, respectively, identified by software analysis of cryo-EM images as apoferritin and lumazine synthase, respectively. The dashed lines indicate 4-fold (**C**) and 2-fold (**D**) axes of rotational symmetry. Panels E and F depict the initial 3D model obtained by single-particle reconstruction of the 15 nm particles, showing the characteristic pentagonal (**E**) and hexagonal (**F**) vertices of an icosahedron. (**G** and **H**) The figures show the refined reconstructions of the particle classes from panels C and D, respectively, with the solved crystal structures of bacterioferritin and lumazine synthase superimposed, shown from the outside (left) and in cross-section (right).

Round or oval EVs with clearly visible lipid bilayers were also observed using cryo-EM in all samples (Fig. 3A). Because there was little evidence of cellular debris apparent in cryo-EM images, we concluded that these were likely the products of EV release by membrane “blebbing” rather than products of lysis. EVs averaged approximately 20 nm in diameter with a range from 8.4 to 130 nm, and EVs obtained from *Alteromonas* evolved at 800 ppm pCO_2_ were slightly (< 1 nm) smaller on average than those from ancestral strains or strains evolved at 400 ppm (linear model, *P* = 0.068 and *P* = 0.023, respectively, Fig. S16). Large granular particles were also observed (Fig. 3B). In two cases, we were able to use averaged cryo-EM images to identify and reconstruct large symmetrical protein complexes from image data. For the subset of 12 nm particles, a model using C4 then D4 symmetry was used for 3D reconstruction (Fig. 3C). For the 15 nm particles (Fig. 3D), an initial model generated with C2 symmetry displayed higher-order icosahedral symmetry, which was applied in subsequent auto-refinement to produce the final reconstruction (Fig. 3E and F). The 12 nm particles fit a model of bacterioferritin (PDB:3E1J) with a correlation of 0.96 (Fig. 3G). A search of icosahedral structures in the Protein Data Bank identified the 15 nm particles as lumazine synthase (PDB:1NQU), which was fit to the reconstruction with a correlation of 0.97 (Fig. 3H). The last two steps of riboflavin biosynthesis are catalyzed by lumazine synthase (RibH and RibE) and are found together as a large 60-mer bifunctional icosahedral complex (53) like the one observed here; both of these proteins were detected in the proteomes of all three supernatant samples (Table S1). Similarly, bacterioferritin forms a large cage-like structure comprised of 24 monomers that functions to sequester Fe atoms (54) and was observed in our proteome. While both complexes are large enough to be retained by a 50 kDa filter, they are also canonically cytoplasmic proteins with no known secretion mechanism. Given the evidence described above that these proteins were not released by simple cell lysis, we suggest that both of these complexes may have been associated with EVs or released through processes like those that led to the formation of EVs.

### Exudates from *Alteromonas* are biologically active and physically associate with *Prochlorococcus* cells

**Fig 4:**
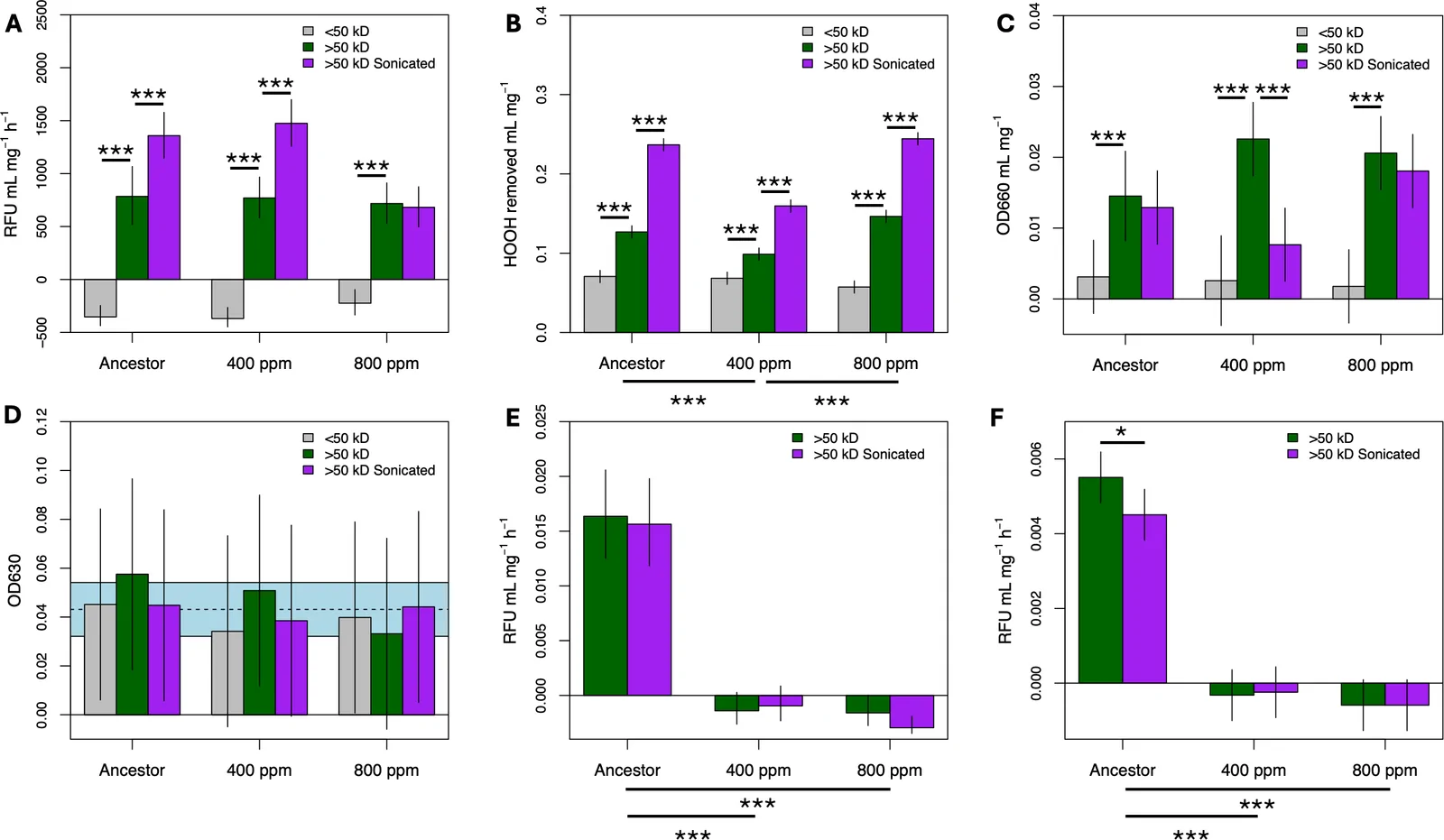
Biological activity in *Alteromonas* exudates. *Alteromonas* strains were isolated from cultures either before (Ancestor) or after 500 generations of evolution at modern or projected future pCO_2_ conditions (400 ppm and 800 ppm, respectively). Cultures were filtered to remove cells and separated into >50 kDa and <50 kDa size fractions by tangential flow filtration. For panels **A** through **D**, enzyme activity was assessed for both fractions as well as for the >50 kDa fraction after a sonication treatment to disrupt vesicles; the <50 kDa fraction was omitted for panels **E** and **F** because the targeted enzymes were expected to be greater than 50 kDa. Readings from sterile media blanks were subtracted from all output variables. Enzyme assays (**A–C and E and F**) were normalized against the total protein concentration of the sample. (**A**) Phosphatase, (**B**) hydroperoxidase (e.g., catalase), (**C**) protease, (**D**) siderophores, (**E**) alpha-glucosidase, and (**F**) NAGase. Error bars reflect the 95% confidence intervals from post-hoc *emmeans* tests of linear models of the impact of strain and treatment on the dependent variable. For panel **D**, no significant differences were detected between experimental groups; hence, we have also included the mean (dashed line) and 95% confidence interval (light blue box) of the combined data to demonstrate that siderophore activity was significantly higher than the blank in these experiments. Asterisks above bars indicate the results of a within-strain Tukey test; asterisks below bars indicate tests of the effect of evolution using the average of all within-strain treatments. *, *P* < 0.05; **, *P* < 0.01; ***, *P* < 0.001.

The detection of catalytic enzymes within exudates by both proteomics and cryo-EM led us to investigate the activity of the >50 kDa supernatant fraction (Fig. 4). Phosphatase (Fig. 4A), hydroperoxidase (e.g., catalase) (Fig. 4B), and protease (Fig. 4C) activity were detected in all supernatants and were much higher in the >50 kDa than the <50 kDa fraction, consistent with high molecular weight enzymes rather than abiotic processes being responsible for these measured activities. Siderophore activity was detectable in all supernatant fractions (Fig. 4D), but surprisingly, it was not significantly different between the size fractions, despite the low molecular weight of siderophore molecules (55). Coupled with the detection of outer membrane siderophore receptors found only in the >50 kDa fraction (e.g., Fiu in Table S1), this suggests that these molecules may have been associated with EVs. We detected α-glucosidase (Fig. 4E) and NAGase (Fig. 4F) activity in supernatants collected from the ancestral strain of *Alteromonas,* but not strains collected from the end of an evolution experiment; β-glucosidase activity, on the other hand, was not detected in any sample. We also measured enzyme activity after a sonication treatment was conducted to disrupt the lipid bilayers, reasoning that if at least some of the enzyme activity was packaged in EV lumens, sonication would release them, and the observed enzyme activities should increase. Significantly increased activities for phosphatase and/or hydroperoxidase were detected post-sonication for all *Alteromonas* strains (Fig. 4A and B).

**Fig 5:**
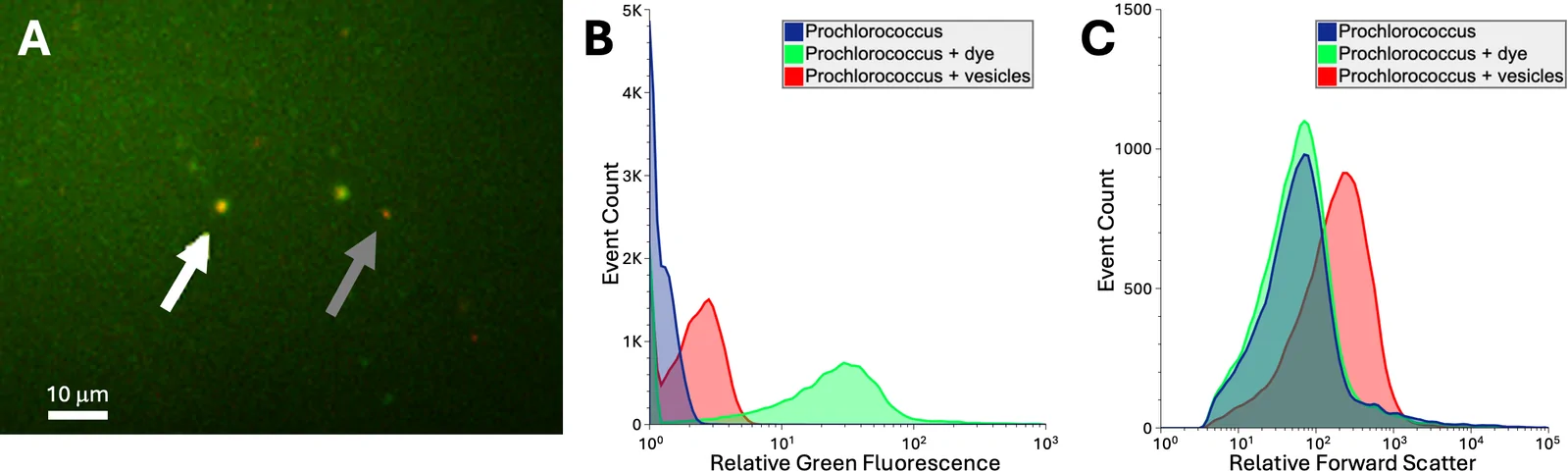
*Alteromonas*-derived exudates physically associate with *Prochlorococcus* cells. (**A**) Exudates labeled with the amine-reactive dye Alexa Fluor 488 were able to stain *Prochlorococcus* cells, suggesting a physical association between exudates and cells. The gray arrow indicates an unstained *Prochlorococcus* cell, appearing red from chlorophyll fluorescence; the white arrow indicates a cell also carrying green fluorescence delivered by a stained vesicle. (**B**) Both the addition of the dye Alexa 488 as well as dyed *Alteromonas* exudate to *Prochlorococcus* cultures marked the cells with green fluorescence. (**C**) In addition to contributing green fluorescence, the addition of dyed exudate (but not Alexa 488 alone) increased the size of *Prochlorococcus* cells as measured by forward light scatter, suggesting aggregation of cells and stained vesicles.

Membrane vesicles are responsible for transportation and delivery of cargo in both prokaryotic and eukaryotic systems (56, 57). We reasoned that *Alteromonas* EVs or other extracellular products in our co-culture system might operate in a similar fashion if they tended to physically associate with *Prochlorococcus* cells, delivering leaky functions between cells in a way that could avoid the problems of dilution and degradation in dilute seawater described above. When concentrates from *Alteromonas* >50 kDa supernatant fractions were stained with a green-fluorescing amine-reactive dye and imaged with *Prochlorococcus* cells, exudates and *Prochlorococcus* were observed to associate (Fig. 5A). Flow cytometry also detected an increase in forward scatter (i.e., size), as well as the appearance of green fluorescence signals for *Prochlorococcus* cells incubated with stained >50 kDa supernatant fractions from all *Alteromonas* strains examined (Fig. 5B–C; Fig. S17). Addition of dye directly to *Prochlorococcus* cells added green signal but did not change the forward scatter profile or chlorophyll fluorescence, suggesting that physical association with exudates rather than transfer of dye to *Prochlorococcus*, and/or exudate-induced aggregation of *Prochlorococcus* cells, occurred in these samples. These results are consistent with the occurrence of direct, physical interactions between *Alteromonas* exudates and *Prochlorococcus* cells when they were incubated together. Moreover, the impact of these associations on the apparent size of the cells is more consistent with aggregation of *Prochlorococcus* with EVs (labeled via covalent attachments between the dye and transmembrane proteins), although we cannot rule out the possibility that other peptides in the exudate also contribute.

### Purified *Alteromonas* EVs enhance *Prochlorococcus* growth

**Fig 6:**
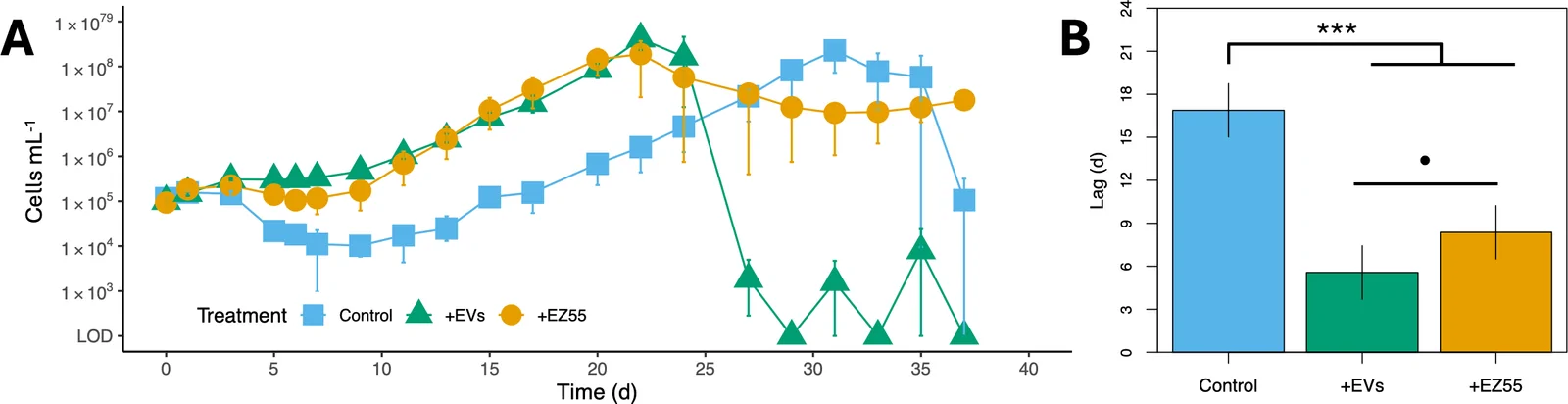
Purified *Alteromonas* EVs enhance *Prochlorococcus* growth. (**A**) *Prochlorococcus* cell densities over time in cultures grown axenically (Control, squares) or with the addition of either purified EVs (triangles) or live *Alteromonas* EZ55 cells (circles). Symbols represent the mean of three replicates, and error bars extend to the maximum and minimum values at each time point. Limit of detection of flow cytometric detection (~100 cells mL^−1^). (**B**) Lag phase duration for the cultures shown in panel A. Error bars represent the 95% confidence intervals of the predicted mean for each treatment group from a linear model. Asterisks indicate the results of pairwise post-hoc extended marginal means comparisons: •, *P* < 0.1; ***, *P* < 0.001.

We next sought to test to what degree EVs specifically conferred the helper phenotype for *Prochlorococcus* cultures. We used a density gradient ultracentrifugation method to separate EVs from other components of the >50 kDa fraction and then added the highly purified EVs to axenic *Prochlorococcus* cultures at an initial concentration of 10^8^ EVs mL^−1^, similar to concentrations that were likely present in our earlier experiments (Fig. 1E). The growth curves of EV-treated cultures were compared to unmodified axenic cultures or cultures containing intact, living *Alteromonas* cells (Fig. 6A). EVs exhibited a clear protective effect at the early stages of growth, significantly reducing the lag duration after culture inoculation (Fig. 6B). However, EVs alone did not continue to exert growth-enhancing characteristics at later stages of the batch culture cycle; EV-only cultures did not have significantly higher maximum cell densities (Fig. S18A) than controls, and cultures with live bacteria grew significantly faster (Fig. S18B) and survived longer after growth cessation (Fig. 6A) than EV-treated cultures. Thus, some, but not all, of the growth-enhancing characteristics of *Alteromonas* exudates are directly associated with EVs. Analysis of EVs recovered from cultures at the end of the experiment revealed changes in EV size compared to the initial EV inoculum (Fig. S19). Additionally, particle concentrations in *Prochlorococcus* cultures treated with EVs were ~50% lower than axenic *Prochlorococcus,* with no EVs added, and significantly lower than the initial EV inoculum (Fig. S20). This suggested to us that most of the initial *Alteromonas* vesicles had either degraded or fused with *Prochlorococcus* cells. It is thus possible that the absence of EV protection in late batch culture may reflect the degradation of EVs over time rather than an absence of protective capacity in the EVs.

### Evidence for genetic control of *Alteromonas* exudation

Finally, we were interested to know if the composition and activity of exudates was under genetic control in *Alteromonas*. To explore this, we compared *Alteromonas* strains isolated before (Ancestor) and after 500 generations of evolution at either modern (400 ppm) or projected year 2100 (800 ppm) pCO_2_ conditions (21). The proteins discovered in the supernatant proteome of *Alteromonas* after evolution with *Prochlorococcus* at 800 ppm were notably different than either the ancestral or 400 ppm evolved lineages (Fig. S12). Of the 120 proteins that were not shared between all three strains, 33% were shared between the ancestor and the 400 ppm strain, whereas only 1.7% and 4.2%, respectively, were shared between the 800 ppm strain and the ancestor or the 400 ppm strain. Although the distribution of protein localizations and functional groups was not notably different between the strains (Fig. 2; Fig. S15), we observed significant differences in enzyme activity, with significantly altered α-glucosidase, NAGase, phosphatase, and hydroperoxidase activity in >50 kDa concentrates from the different *Alteromonas* strains (Fig. 4). As mentioned above, a small but significant change in EV size was also measured in *Alteromonas* evolved at 800 ppm (Fig. S16A). Each of these strains was grown under identical conditions; hence, these reproducible changes likely reflect genetic differences that arose during evolution (21), suggesting that protein secretion, cellular leakage, and/or vesicle packaging and release were controlled by genetic mechanisms for these organisms. These observations are consistent with evolutionary changes in *Alteromonas*’ helping ability observed previously (21), although the present results do not allow us to connect those changes to particular proteins or functions, or to EVs specifically.

## DISCUSSION

Here, we show evidence that *Alteromonas* facilitated greater exploitation of medium resources by *Prochlorococcus*, allowing them to grow to greater cell densities, delay growth cessation, and avoid cell death in batch co-cultures, potentially by degrading autotoxic substances produced in dense *Prochlorococcus* cultures. Similar results were previously observed for both *Prochlorococcus* (20) and its sister taxon *Parasynechococcus* (58), where the presence of heterotrophic bacteria prolonged survival in long-term cultures. We extend these observations to show that this facilitation is largely accomplished by high-molecular weight heat-labile exudates of *Alteromonas* exhibiting diverse biological activities and that some of these products may be packaged within EVs. Facilitation of this sort is a central mechanism underlying the BQ Hypothesis that allows reductive genomic evolution and the development of complex exchanges between strains and species in microbial communities (2, 3). This work suggests that EV and protein release are components of this process, at least for the *Prochlorococcus/Alteromonas* interaction. Several previously described BQ functions were observed and/or indicated in cell-free filtrates here, including H_2_O_2_ removal (11, 18), Fe sequestration (6, 59), vitamin biosynthesis (60, 61), and polymer degradation (9, 62–64).

We cannot attribute facilitation exclusively to EVs since fully extracellular proteins were also observed (Fig. 3) and since purified EVs alone did not fully reconstitute the helping ability of living *Alteromonas* cells (Fig. 6). Moreover, many of the putative EV-associated enzymes whose activities we measured (e.g., phosphatase and catalase; Fig. 4) were large enough to have been retained by the 50 kDa filter without being contained in EVs (Table S1), and we observed at least two large extracellular protein complexes by cryo-EM as well (Fig. 3). It is also unclear to what degree these products were actively packaged as opposed to assorted into EVs based on inherent biases in their subcellular location or other biochemical properties or if instead they were released through uncontrolled processes such as lysis or imperfect cell division. To our knowledge, there are no reports of bacterioferritin or lumazine synthase secretion or any established mechanisms by which such large complexes could be released from healthy cells. However, the broadly different protein profiles of lysed cells compared to supernatants (Fig. S13) argued strongly against simple lysis explaining our results, and evidence exists in many bacterial species that diverse proteins, including large multimers, are secreted through unknown, non-classical mechanisms, including via EVs (65). Moreover, shifts observed in extracellular proteins following evolution (Fig. 4; Fig. S12) suggested at least some level of regulation acting on protein export. The apparent loss of α-glucosidase and NAGase activity from evolved *Alteromonas* exudates (Fig. 4E and F), for instance, is consistent with adaptive evolution, since neither starch nor chitin are produced by *Prochlorococcus*, rendering these enzymes superfluous under the conditions of the LTPE. Evolutionary changes in bacterial EV characteristics have previously been observed in the Lenski long-term *Escherichia coli* evolution experiment (66), and studies have suggested genetic mechanisms contributing to EV production, secretion, and packaging (67, 68), indicating that EV composition may be a target of selection during bacterial adaptation. Experiments with evolved *Alteromonas* isolates should be able to identify genetic determinants of altered EV structure and function and improve our understanding of the packing and release of EVs in marine gram-negative bacteria.

The presence of EVs raises interesting possibilities for the interaction between *Prochlorococcus* and other bacteria specifically, and for BQ interactions more generally. One objection against passive BQ interactions being important in the open ocean is that the average distance between a *Prochlorococcus* cell and the nearest heterotrophic bacterium may be hundreds of cell diameters on average (69), and if the leaked products are simply excreted into the surrounding environment, their concentrations will exponentially attenuate with distance and will be dramatically diluted by the time they reach potential beneficiaries. Whether the leaked products were metabolites or enzymes, dilution would greatly reduce their impact on their neighbors (69). EVs address this problem in two ways. First, EVs protect secreted materials from degradative environmental factors such as ultraviolet light, suboptimal chemical conditions, and extracellular enzymes, increasing the effective range that secreted materials may disseminate while retaining their proper form and function (68, 70). Second, EVs provide a means to keep secreted products close to beneficiary cells once they encounter each other, as they appear to establish stable aggregations with cells (Fig. 5) where fully dissolved proteins may instead remain randomly distributed. Additionally, some fraction of EVs may stay within the diffusive boundary surrounding cells (71), further helping spatially constrain the shared functions and maintain a locally high relative concentration.

It is also possible that, rather than simply associating with beneficiary cells, EVs instead fuse with the outer membrane, releasing their contents into the periplasm. EV attachment, followed by membrane fusion, has been proposed as a mechanism for bacterial EVs to deliver compounds to target cells (72–74). Our observations do not preclude the possibility that *Alteromonas* EVs fuse in a similar fashion with *Prochlorococcus* cells. If such events occurred, it would give *Prochlorococcus* at least temporary access to a much wider variety of proteins than its meager genome encodes. While EVs have previously been implicated in genetic transfers between bacteria (75, 76), the interspecies transfer of active enzymes would reflect a form of horizontal information transfer and suggest a wide expanse of possibilities in understanding metabolism, communication, and cooperation among bacteria. Until the specific mechanisms underlying cell/EV association are uncovered, our ability to translate our observations to an explicit molecular model of facilitation will remain limited. Future efforts (e.g., using isotope or nanoparticle labeling of vesicle lipids or contents) should address the ultrastructural nature of these interactions and the ultimate fates of EVs and their cargoes in this system.

Another intriguing possibility is that EVs may preferentially associate with particular cells, either of the same species that generated the EVs or with other partners. There is some experimental evidence that this occurs in nature (39, 77) and after laboratory evolutionary differentiation (66), but mechanistic explanations are lacking. While our study was not designed to test this hypothesis, our data nevertheless highlight possible mechanisms for specifying EV delivery. Our proteomic results include numerous proteins predicted to span the outer membrane, including various receptors and transporters (Table S1) that could potentially recognize structures on the outside of other cells or otherwise influence interactions through alterations in surface charge or hydrophobicity. Moreover, we show evidence that *Alteromonas* exudates, EV size, and possibly also EV packaging changed during the adaptive evolution of *Alteromonas* strains to altered pCO_2_ conditions (Fig. 4; Fig. S16). Previous work also suggested convergent evolution of cell surface features in *Alteromonas* and *Prochlorococcus* strains evolved alongside each other (21). Under the simplest formulation of the BQ Hypothesis, leaky functions yield *public* goods that are freely available to the entire community, but if these functions were packaged, as in EVs, in a manner that could be targeted to specific recipients, these would become *club* goods (i.e., available only to recipients who possessed appropriate credentials in the form of cell surface receptors) and would add another layer of complexity to the evolution of microbial metabolic markets. Future work should investigate the mechanism underlying the physical association between EVs and cells in this system.

While our results show that enzymatically active *Alteromonas* exudates alter Pro99 media in a way that improves *Prochlorococcus* growth and survival under high-density culture conditions, it remains unclear what activity is responsible for this facilitation. Our experiments with conditioned media strongly suggest that at least one compound secreted by *Prochlorococcus* caused growth cessation and cell death in Pro99 cultures and that this compound was deactivated or degraded by *Alteromonas* exudates (Fig. 1; Fig. S9), similar to results previously observed by others (20, 58). Several other studies of spent *Prochlorococcus* culture media have revealed a wide variety of metabolites (78, 79), as well as high exudation rates in post-growth cultures (80), but none were obviously toxic. *Prochlorococcus* secretes several organic acids, but they were not sufficiently concentrated to overcome the alkalinizing effect of the photosynthetic carbon concentrating mechanism (Fig. S6) and were therefore unlikely to be able to cause intracellular acid stress. Another possibility is that one or more extracellular compounds signal *Prochlorococcus* to undergo programmed cell death (PCD). PCD-like activity has been observed in natural populations of *Prochlorococcus* and *Synechococcus* following a defined diel pattern (81) and has also been seen in numerous other species of cyanobacteria and algae (82–89). If *Prochlorococcus* does undergo PCD after reaching a certain cell concentration, this suggests two important questions for further investigation: what benefit does it gain from limiting its own growth while leaving unexploited nutrients in the medium, and what benefit does *Alteromonas* gain from staving this process off for a few more generations? Future efforts using targeted gene knockouts or chemical fractionation may provide evidence of the identity of the toxic molecule(s) and further directions for investigation.

A notable limitation of the present study is that most of the observations were collected under conditions that do not closely resemble those in the oligotrophic gyres where *Prochlorococcus* is normally found. The nutrient concentrations in Pro99 are orders of magnitude higher than those in the open ocean, and cell densities in these experiments ranged from approximately 10^7^–10^8^ cells mL^−1^, whereas maximum cell densities in the ocean do not generally exceed 10^5^ cells mL^−1^ (90). Moreover, *Prochlorococcus* densities in the ocean are likely kept below their carrying capacity by grazing mortality (91), leading to continuous nutrient-limited growth rather than the boom-and-bust dynamics of batch culture. The composition and concentrations of *Alteromonas* exudates are likely different under these high-density conditions, potentially magnifying the impact of the mechanisms uncovered in this work. Therefore, while expanding these results into an ecological context is challenging, future work investigating production and activity of *Alteromonas* extracellular products, including EVs, and their impact on *Prochlorococcus* under continuous culture conditions may better clarify their role in native marine processes. Also, because *Alteromonas* is only one of many taxa identified as helpers of *Prochlorococcus*, future work should investigate to what degree exudates and EVs from other lineages have similar impacts on *Prochlorococcus* growth and also how these processes operate in more complex communities.

In conclusion, *Alteromonas* secretes products that reduce *Prochlorococcus* mortality and allow several more generations of growth in Pro99 medium, and some of these products are associated with EVs. The possibility that the proteins responsible for the BQ interaction between *Prochlorococcus* and *Alteromonas* are packaged in EVs opens new possibilities for the evolution of these dynamics, including the possibility that the benefits from leaky functions may be targeted to specific community members instead of the community as a whole, making such benefits club goods instead of public goods in economic parlance. Future laboratory experiments should investigate this possibility in this and other systems, and modeling efforts should explore the range of impacts on community evolution and the origin of mutualisms for such targetable leaky functions.

## Data Availability

The raw data used for this project are available from the BCO-DMO repository, including flow cytometry files (doi: 10.26008/1912/bco-dmo.994735.1), growth curve (doi: 10.26008/1912/bco-dmo.995113.1), enzyme activity measurements (doi: 10.26008/1912/bco-dmo.997078.1), and raw and processed proteomics data (doi: 10.26008/1912/bco-dmo.986127.1).

## SUPPLEMENTAL MATERIAL

Supplemental figures aem.00798-26-s0001.pdf Fig. S1 to S20.

Table S1 aem.00798-26-s0002.csv Peptides identified in culture supernatants or lysates of *Alteromonas*.

ASM does not own the copyrights to Supplemental Material that may be linked to, or accessed through, an article. The authors have granted ASM a non-exclusive, world-wide license to publish the Supplemental Material files. Please contact the corresponding author directly for reuse.
