# Supplementary material for: Vesicle-associated exudates from Alteromonas enhance growth and survival of Prochlorococcus in batch culture
Source: Appl Environ Microbiol. 2026 Aug 4;92(9):e00798-26. doi: 10.1128/aem.00798-26 (PMC13599535; doi:10.1128/aem.00798-26)
Supplement: Supplementary file 1 [file aem.00798-26-s0001.pdf]

Supplemental Material for:

Vesicle-associated exudates from *Alteromonas* enhance growth and survival of *Prochlorococcus* in batch culture

Zhiying Lu[1], Sydney Plummer[1], James Kizziah[2], Steven J. Biller[3], and J. Jeffrey Morris[1]*

[1] Department of Biology, University of Alabama at Birmingham
[2] Department of Microbiology, University of Alabama at Birmingham
[3] Department of Biological Sciences, Wellesley College
* Corresponding author

Materials included:

Figures S1 – S20

Table S1 is provided as a .csv file for download

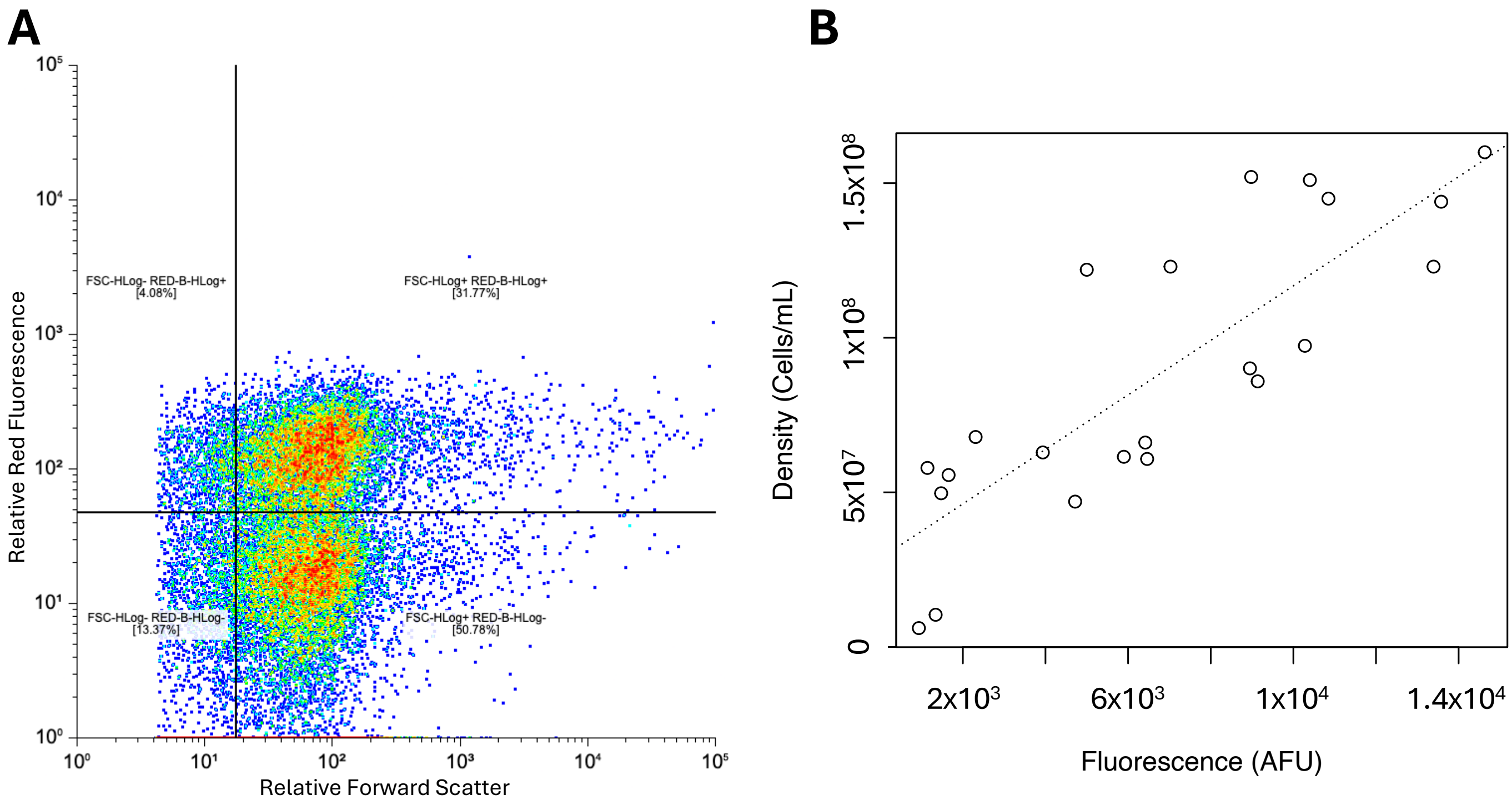

Supplemental Figure 1. Estimation of *Prochlorococcus* cell density. (A) *Prochlorococcus* cultures were enumerated using flow cytometry with gates set on forward scatter (FSC-HLog) and red fluorescence (RED B-HLog) as shown. Events in the upper right quadrant were selected as *Prochlorococcus*. Values in quadrants reflect the percentage of total events contained within the quadrant. **(B)** Some cultures were tracked by bulk chlorophyll fluorescence, which was a robust linear predictor of cell density within the ranges and conditions employed in these experiments. Dashed line, regression of density on fluorescence for cultures simultaneously assessed for each during growth under identical conditions. AFU, arbitrary fluorescence units.

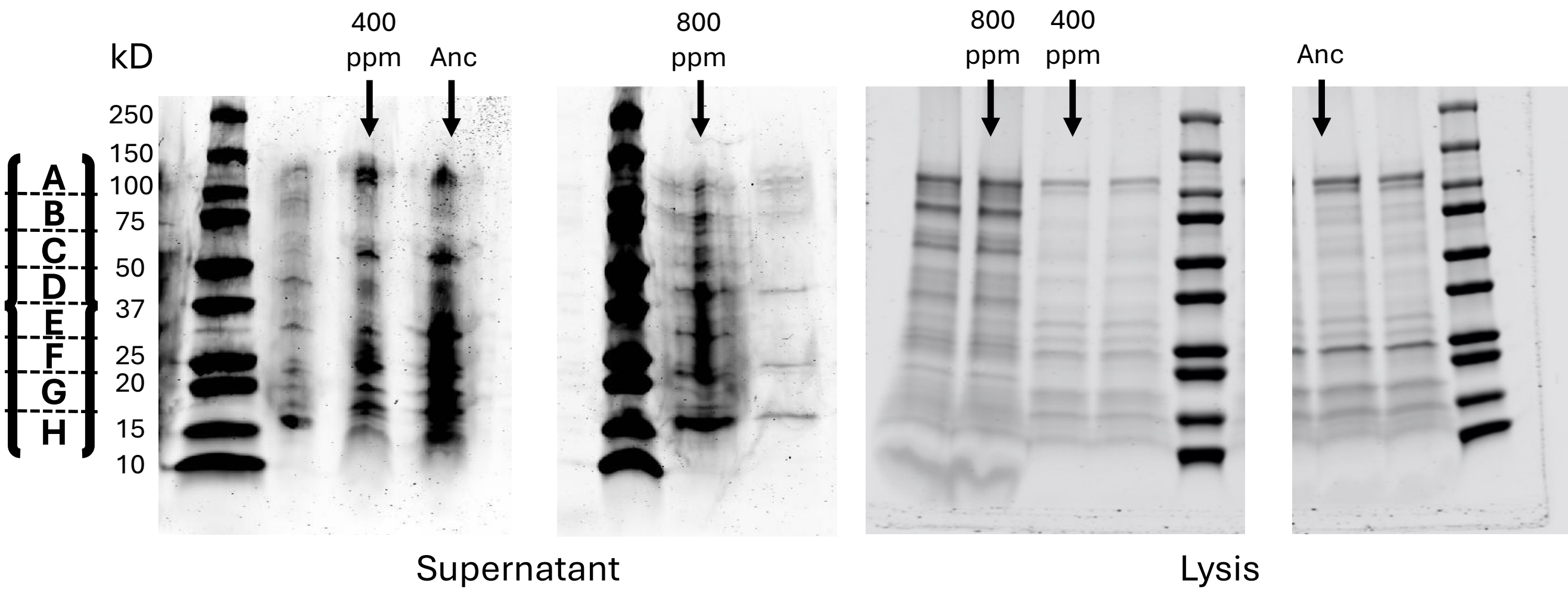

Supplemental Figure 2. Extracted proteins of *Alteromonas* strains before and after evolution. *Alteromonas* strains were isolated from cultures either before (Ancestor) or after 500 generations of evolution at modern or projected future pCO_2 conditions (400 ppm and 800 ppm, respectively). Supernatant proteins were obtained by tangential flow filtration with a 50kD filter; lysis proteins were obtained using a cell lysis kit without further concentration. The A-H scheme on the left hand side of the figure shows the approximate locations of the gel slices that were used for LC-MS peptide identification.

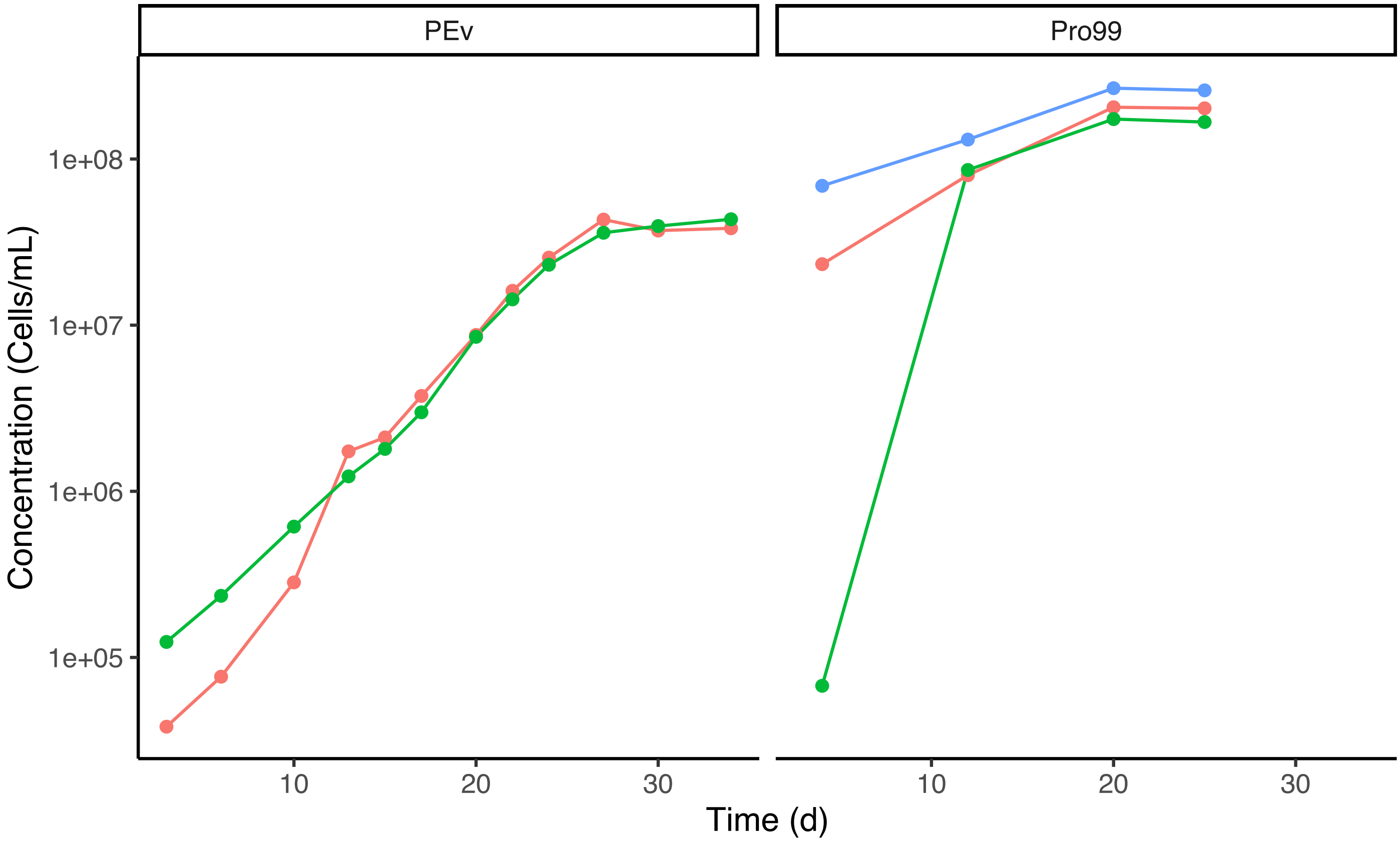

Supplemental Figure 3. *Prochlorococcus* growth in different media. *Prochlorococcus* growth was tracked by flow cytometry for approximately one month during growth at low (~30 mmol photons m^{-2} s^{-1}) light in either PEv or Pro99 media. Different color lines represent different replicate cultures.

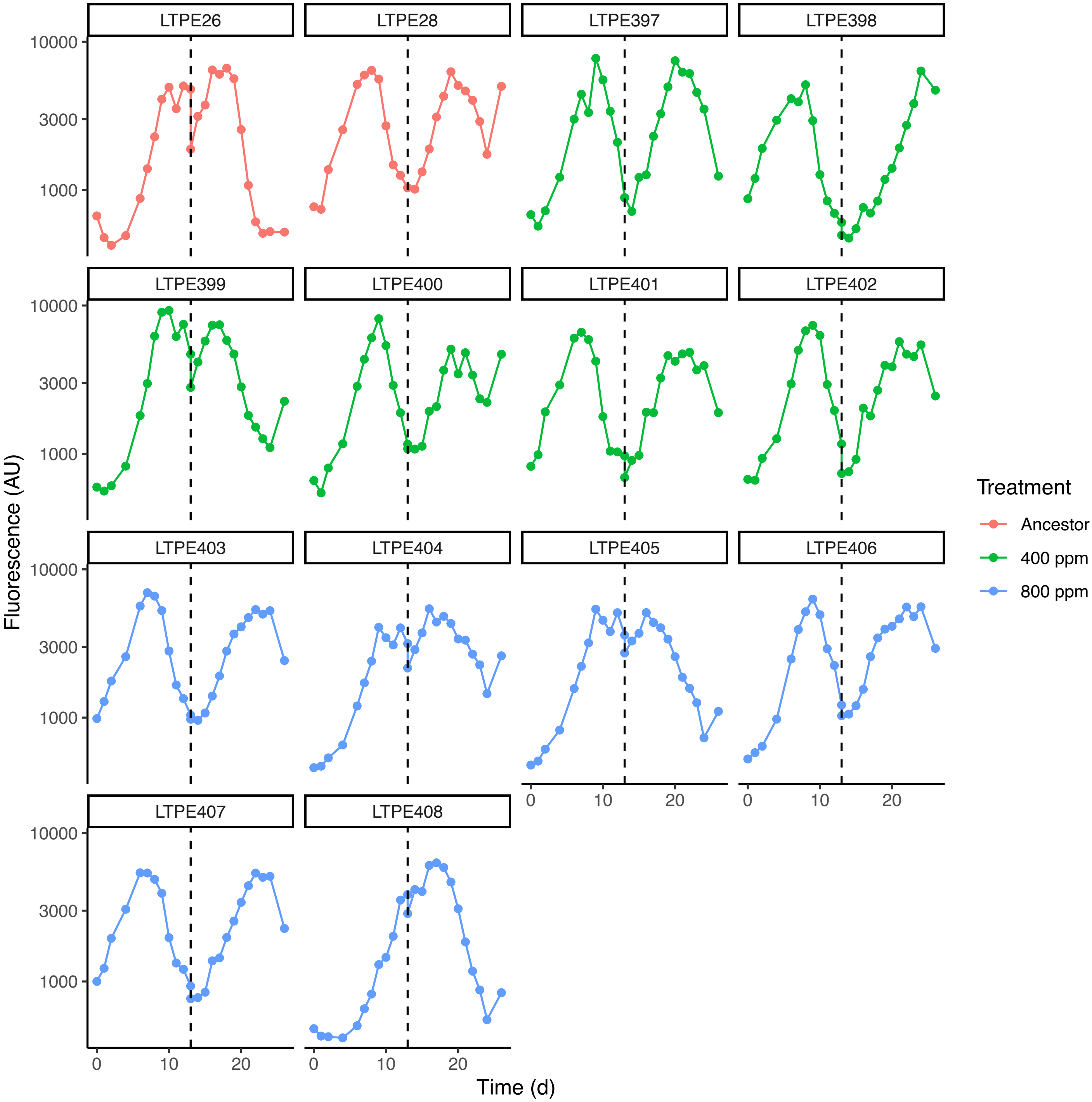

Supplemental Figure 4. Growth of *Prochlorococcus/Alteromonas* co-cultures after dilution into Pro99 medium. Cultures were allowed to grow until fluorescence values began to decline and then diluted 2-fold into fresh Pro99 medium at the time point indicated by the dashed line. Each panel represents a replicate culture from the LTPE experiment with evolutionary treatment indicated by color.

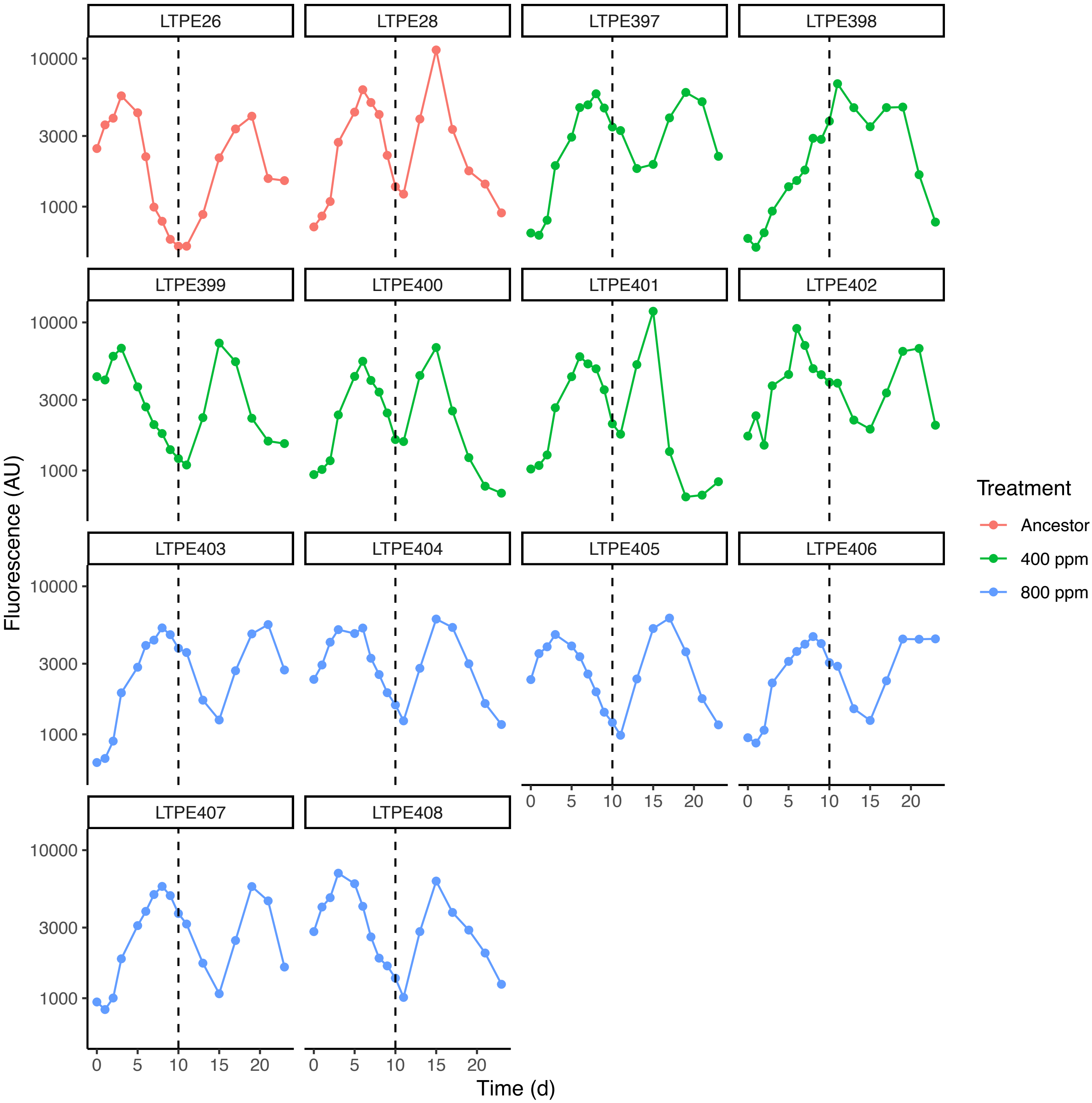

Supplemental Figure 5. Growth of *Prochlorococcus/Alteromonas* co-cultures after dilution into nutrient-free ASW. Cultures were allowed to grow until fluorescence values began to decline and then diluted 2-fold into ASW without added nutrients at the time point indicated by the dashed line. Each panel represents a replicate culture from the LTPE experiment with evolutionary treatment indicated by color.

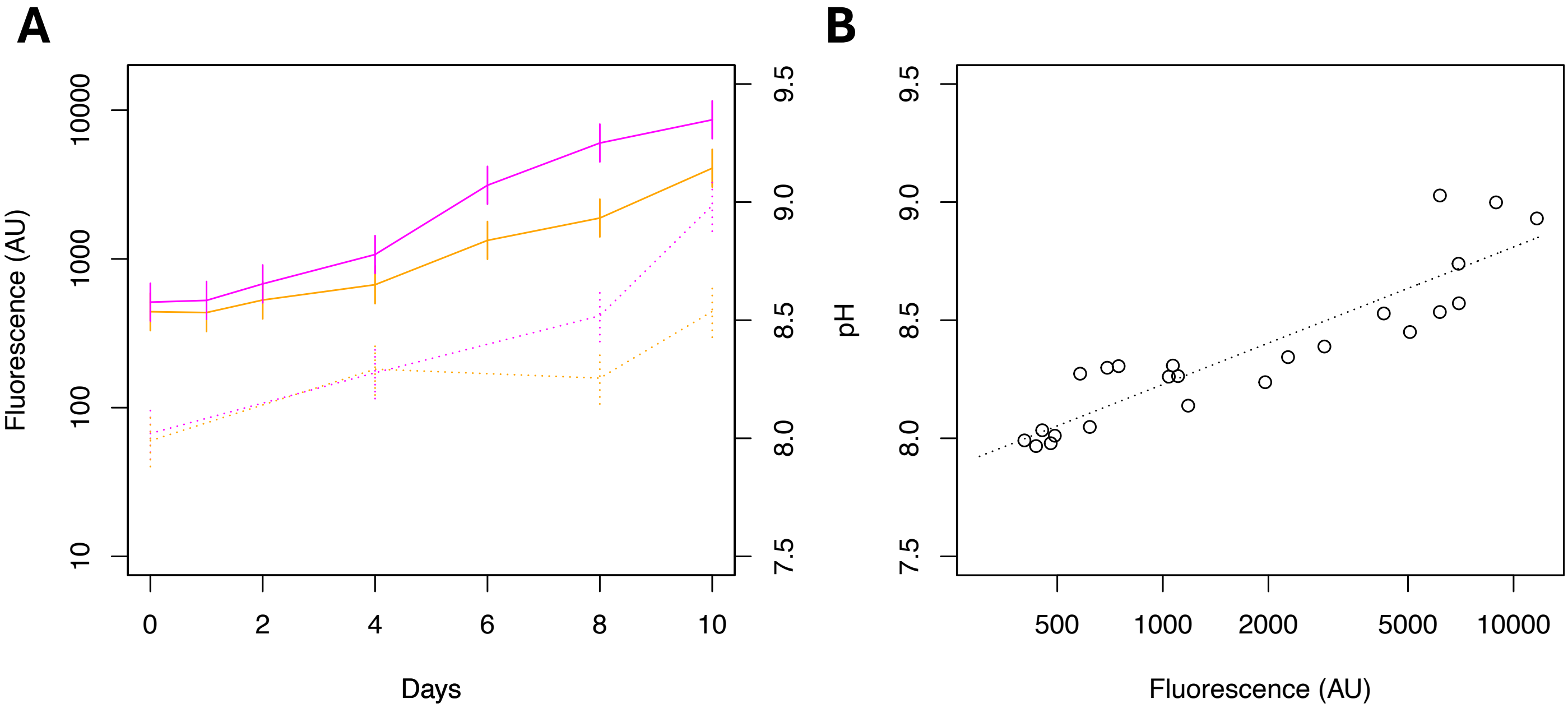

Supplemental Figure 6. Impact of *Prochlorococcus* growth on medium pH. (A) Growth of *Prochlorococcus* (solid lines, left axis) and medium pH (dotted lines, right axis) were measured over the course of a batch culture for three replicates each of LTPE31 (ancestor) and LTPE405 (evolved at 800 ppm pCO_2). **(B)** Log cell density and medium pH were linearly related over the ranges observed in these experiments (slope of regression of pH on log fluorescence 0.253, $r^2 = 0.81$, $p = 1.31 \times 10^{-9}$).

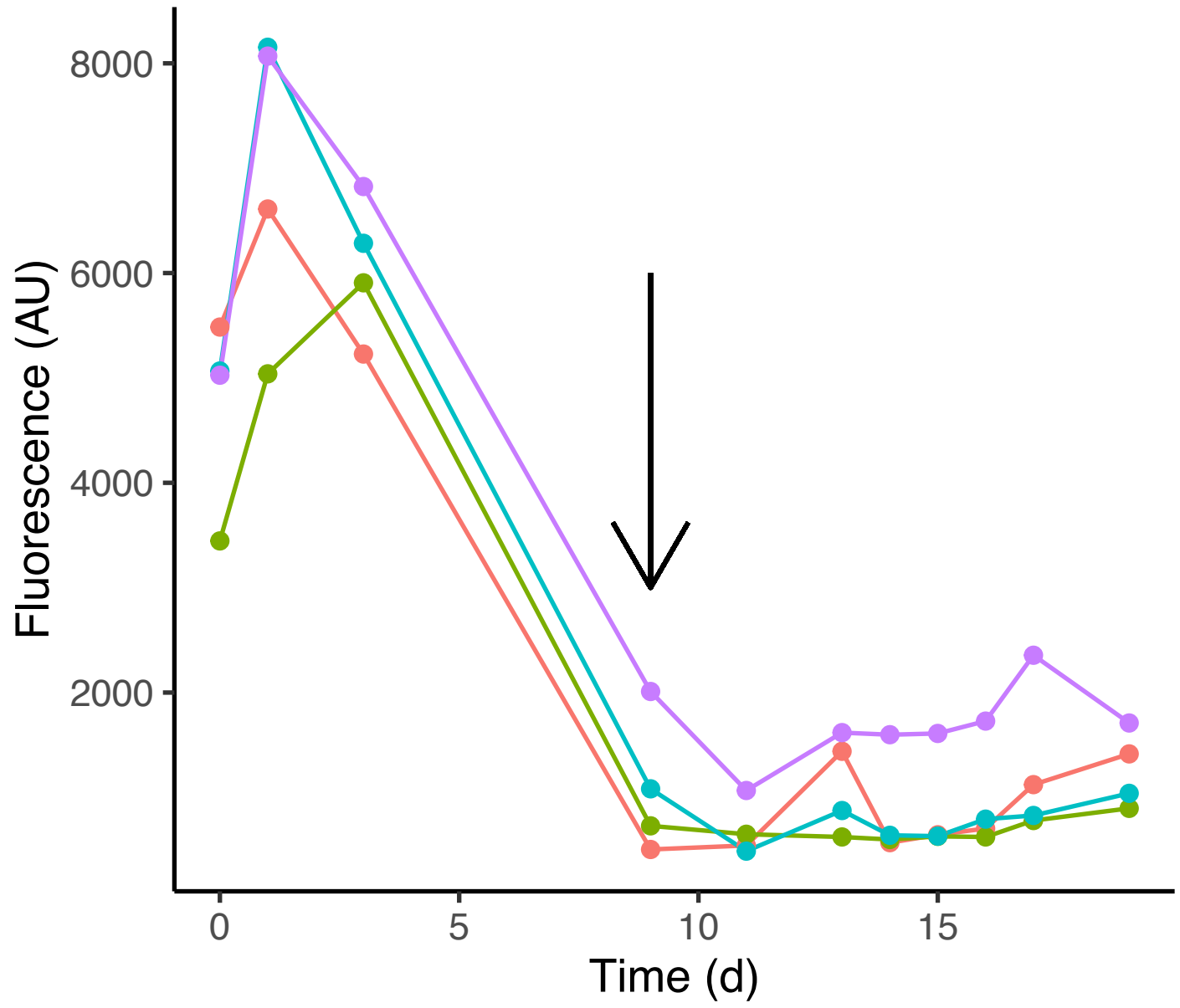

Supplemental Figure 7. *Prochlorococcus* growth cessation is not caused by carbon limitation in Pro99. *Prochlorococcus* was allowed to grow in Pro99 until fluorescence values began to drop, then $NaHCO_3$ was added (at the time point indicated by the arrow). Different color lines represent different replicate cultures from the LTPE experiment.

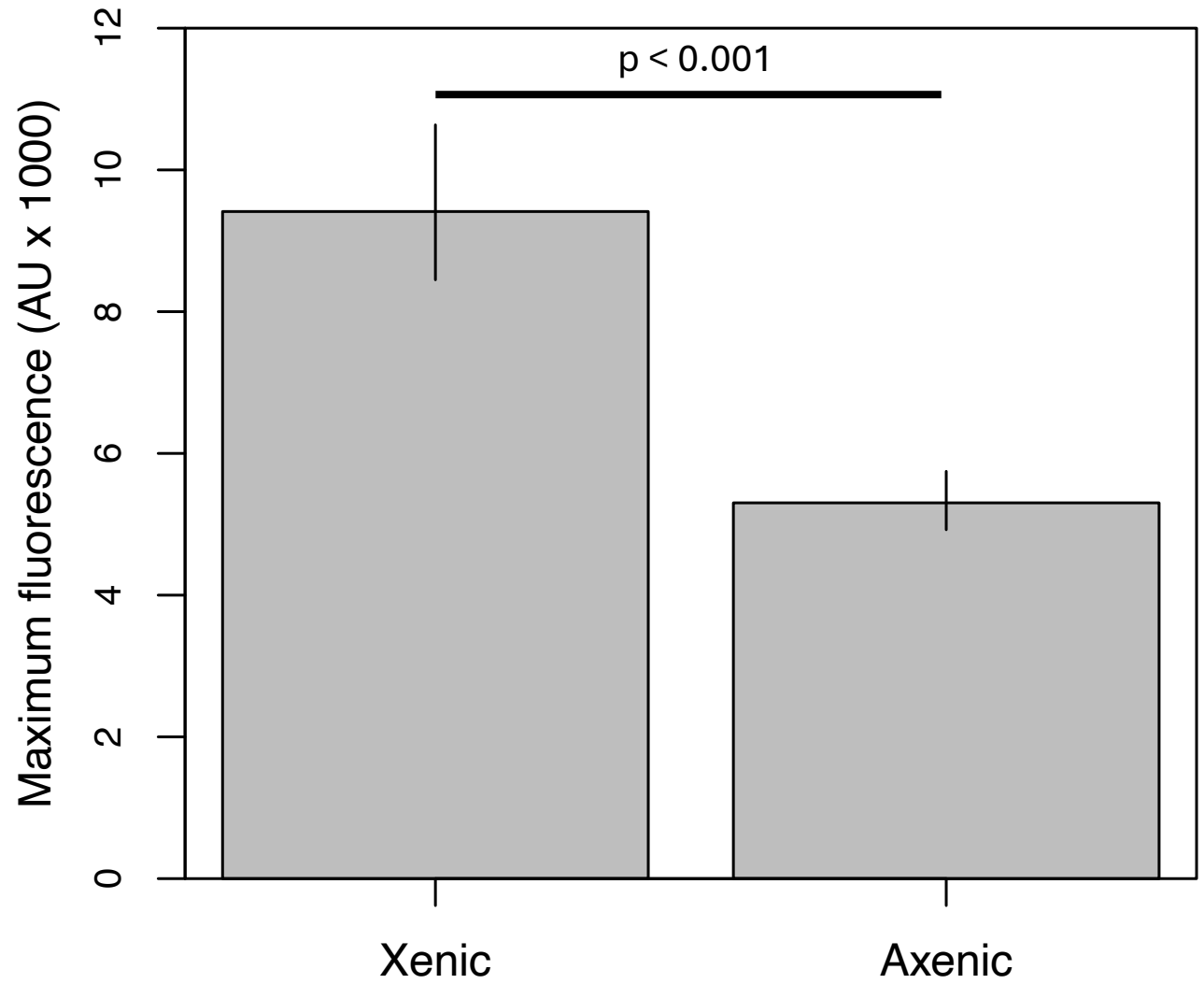

Supplemental Figure 8. Impact of *Alteromonas* on *Prochlorococcus* carrying capacity. *Prochlorococcus* cultures either with (Xenic) or without (Axenic) *Alteromonas* were monitored until growth ceased and began to decline in order to observe their maximum achieved fluorescence (a proxy for total cellular biomass). Axenic cultures ceased growing at a significantly lower cell density than co-cultures.

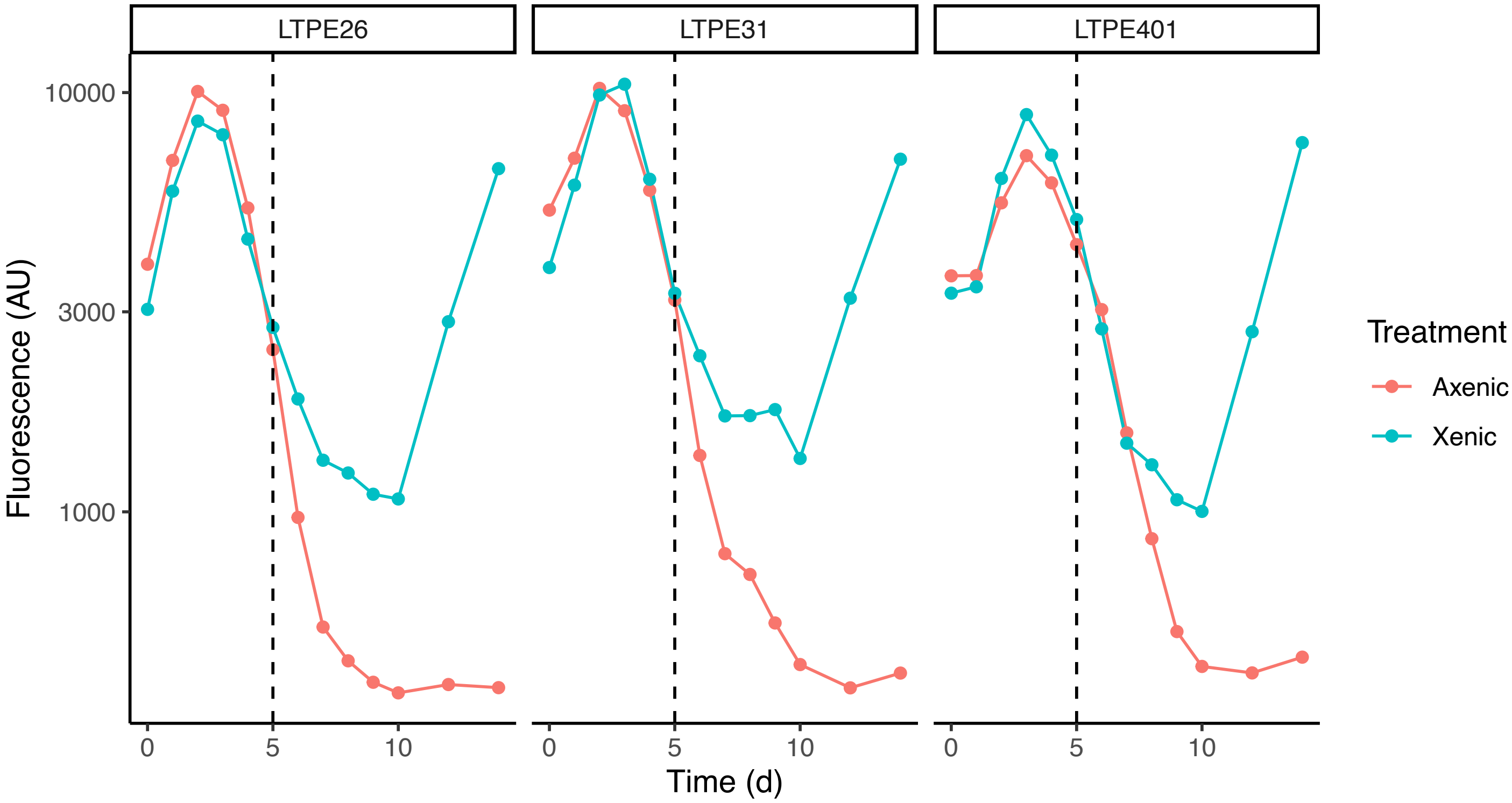

Supplemental Figure 9. Impact of *Alteromonas* on *Prochlorococcus* survival after transfer. *Prochlorococcus* was grown in co-culture with *Alteromonas* until fluorescence values began to drop from their peak. Cultures were then diluted 2-fold into ASW without added nutrients at the time point indicated by the dashed line, either with (Axenic) or without (Xenic) the addition of streptomycin to kill *Alteromonas*. Each panel represents a replicate culture from the LTPE experiment; LTPE26 and LTPE31 were ancestral cultures and LTPE401 was from a culture evolved at 400 ppm pCO_2.

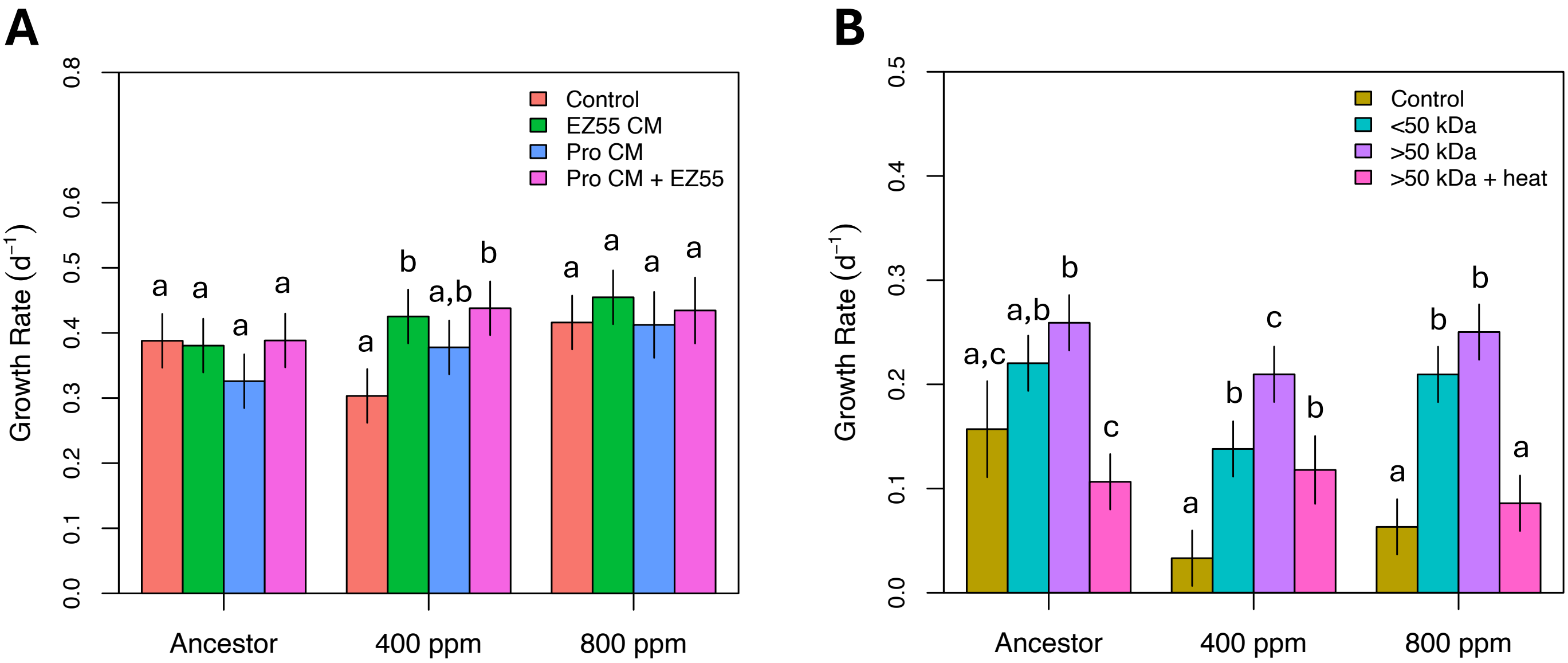

Supplemental Figure 10. *Prochlorococcus* growth rates following treatment with *Alteromonas* exudates. Panels **(A)** and **(B)** reflect the exponential growth rates of the *Prochlorococcus* cultures depicted in Main Text Figures 1A and 1B, respectively. Letters reflect significance groups from a within-strain pairwise Tukey test. Ancestor, 400 ppm, and 800 ppm reflect *Prochlorococcus* MIT9312 strains before or after 500 generations of evolution under altered pCO_2 conditions.

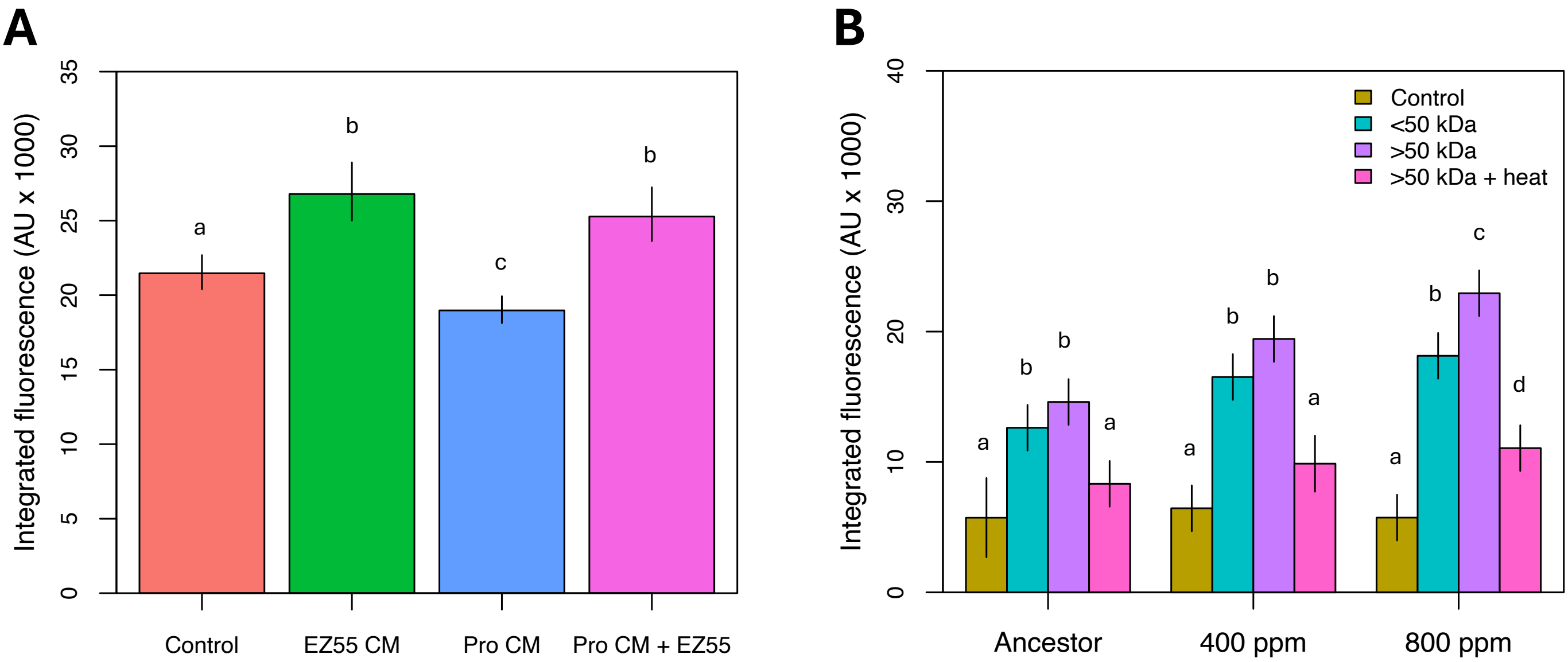

Supplemental Figure 11. *Prochlorococcus* integrated abundance following treatment with *Alteromonas* exudates. Panels **(A)** and **(B)** reflect the area under the growth curve of the *Prochlorococcus* cultures depicted in Main Text Figures 1A and 1B, respectively. Letters reflect significance groups from a within-strain pairwise Tukey test. Ancestor, 400 ppm, and 800 ppm reflect *Prochlorococcus* MIT9312 strains before or after 500 generations of evolution under altered pCO_2 conditions. In panel (A) there was no significant difference between strains, so the linear model only considered treatment.

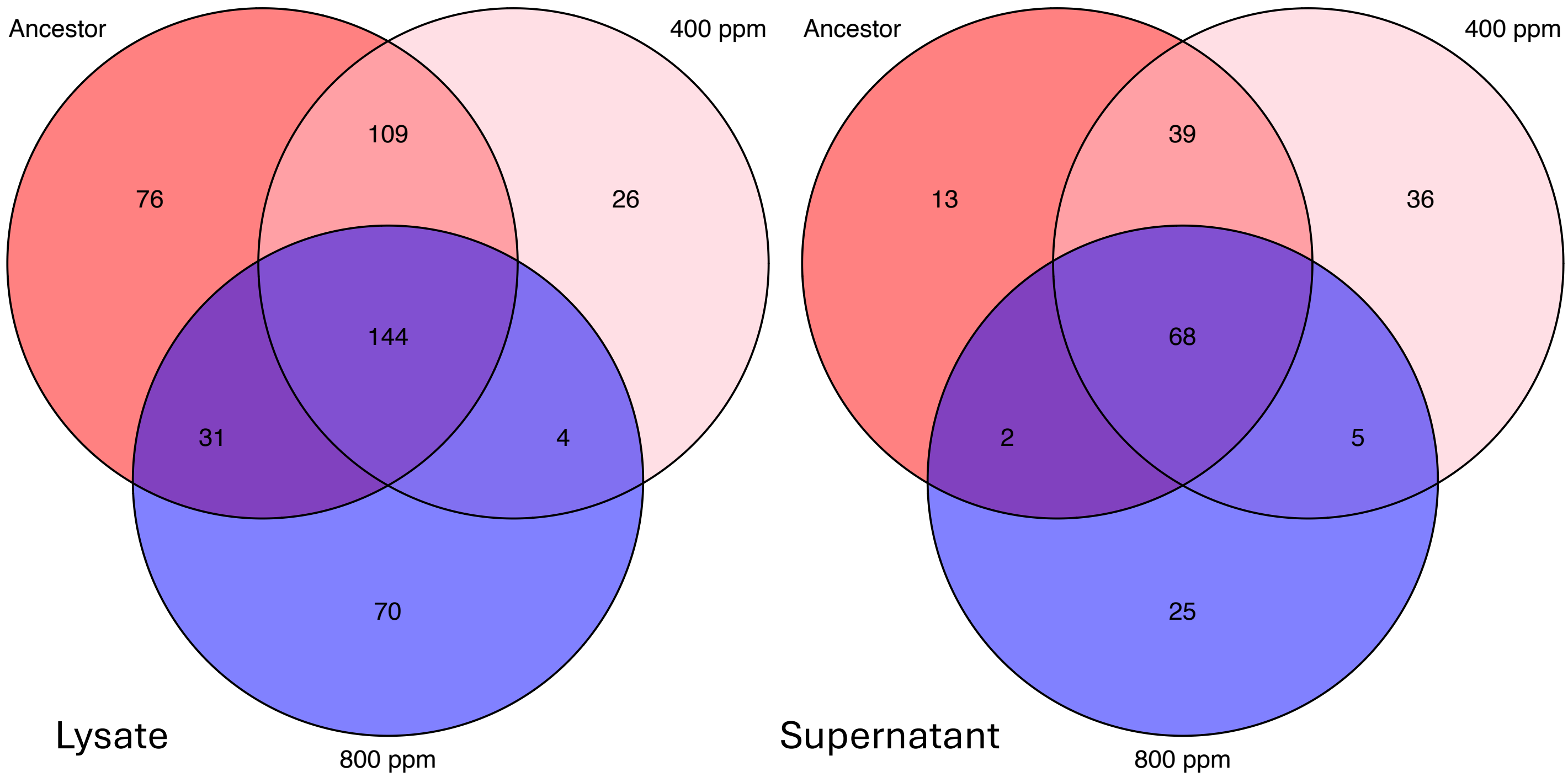

Supplemental Figure 12. Proteome of *Alteromonas* strains before and after evolution. *Alteromonas* strains were isolated from cultures either before (Ancestor) or after 500 generations of evolution at modern or projected future pCO_2 conditions (400 ppm and 800 ppm, respectively). Proteins were identified by LC-MS from whole cell lysates or from concentrated cell-free supernatants. These Venn diagrams show the number of shared proteins between strains for each preparation treatment .

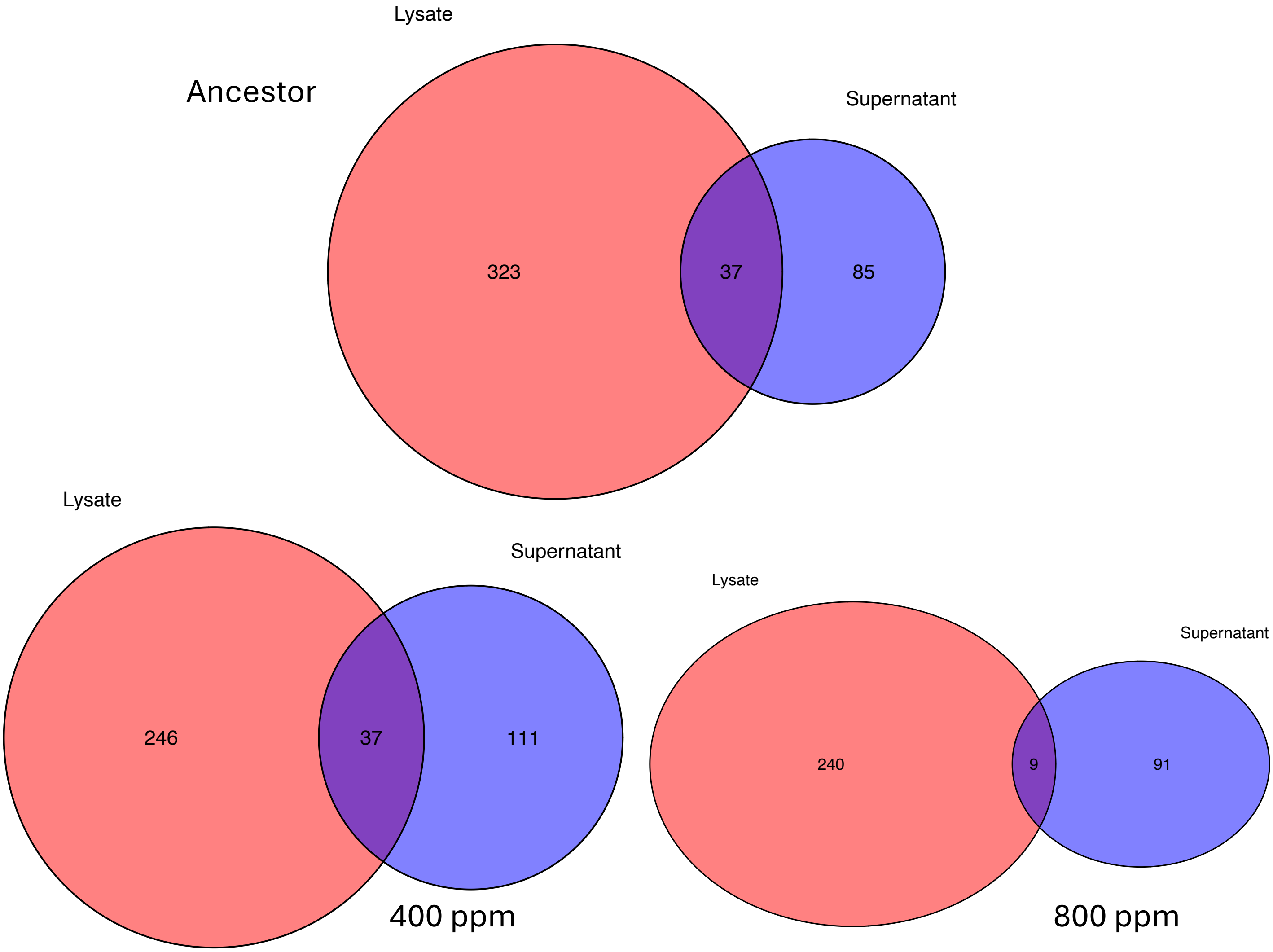

Supplemental Figure 13. Overlap of *Alteromonas* intracellular and extracellular proteomes. *Alteromonas* strains were isolated from cultures either before (Ancestor) or after 500 generations of evolution at modern or projected future pCO_2 conditions (400 ppm and 800 ppm, respectively). Proteins were identified by LC-MS from whole cell lysates or from concentrated cell-free supernatants. These Venn diagrams show the shared proteins between intracellular (lysate) and extracellular (supernatant) treatment groups for each type of strain.

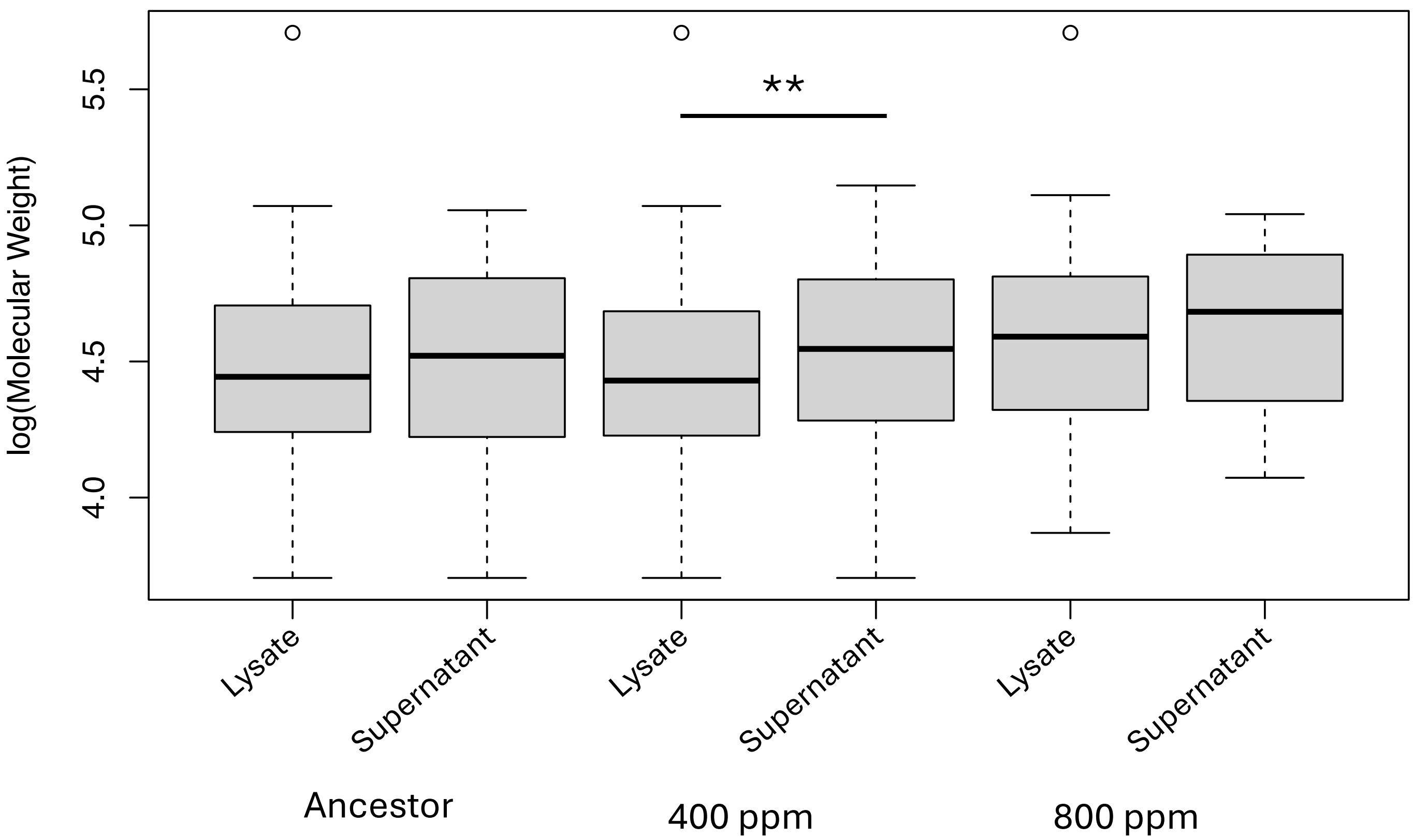

Supplemental Figure 14. Molecular weights of proteins from *Alteromonas* exudates. *Alteromonas* strains were isolated from cultures either before (Ancestor) or after 500 generations of evolution at modern or projected future pCO_2 conditions (400 ppm and 800 ppm, respectively). Proteins were identified by LC-MS from whole cell lysates or from concentrated cell-free supernatants and molecular weights were predicted using corresponding sequences from the *Alteromonas* EZ55 genome. Asterisks indicate $P < 0.01$ from of a post-hoc t-test comparing the mean molecular weight of lysate and supernatant proteins for each EZ55 strain category.

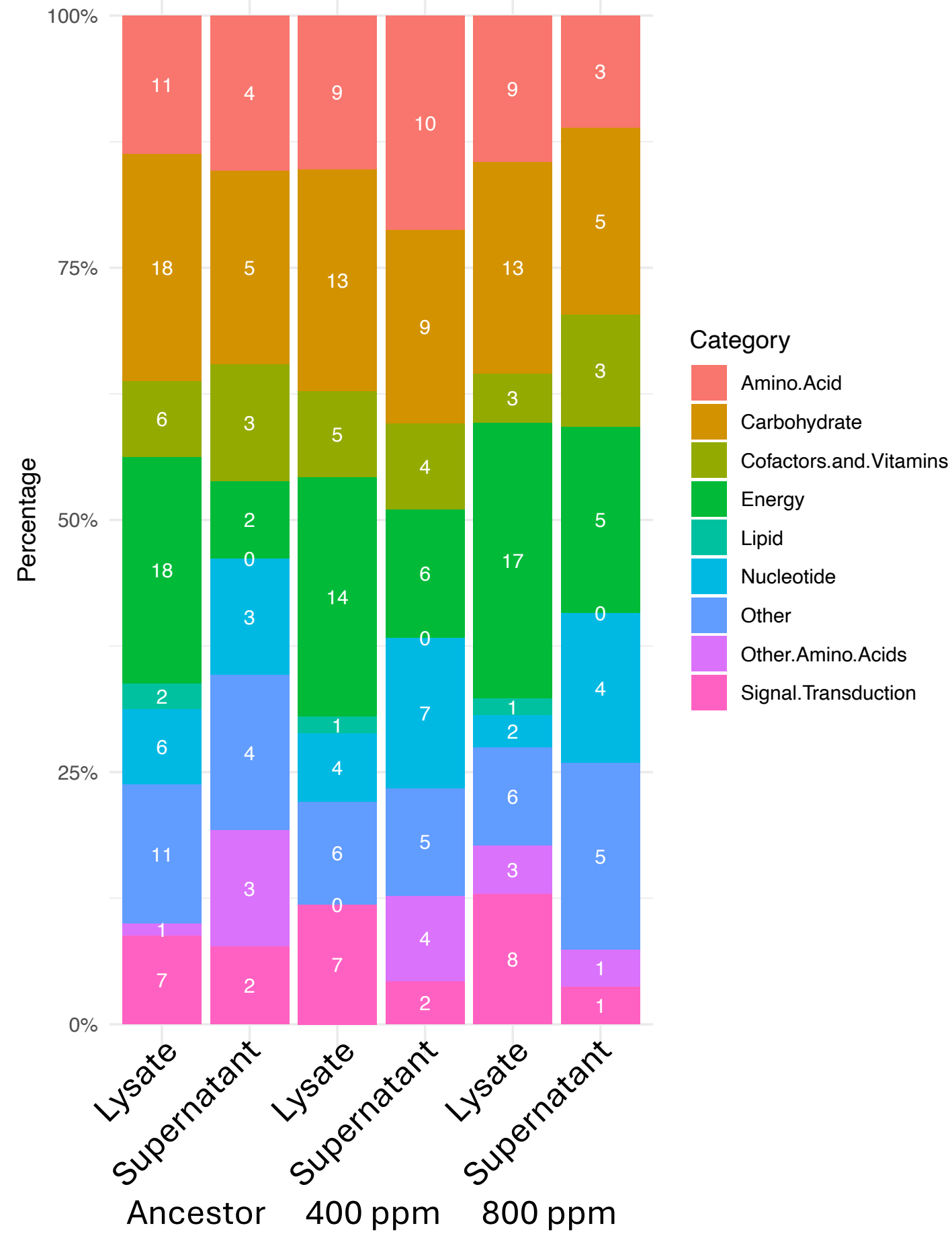

Supplemental Figure 15. Function of *Alteromonas* proteins. *Alteromonas* strains were isolated from cultures either before (Ancestor) or after 500 generations of evolution at modern or projected future pCO_2 conditions (400 ppm and 800 ppm, respectively). Proteins were identified by LC-MS from whole cell lysates or from concentrated cell-free supernatants. Discovered proteins were binned into functional categories using BLASTKoala.

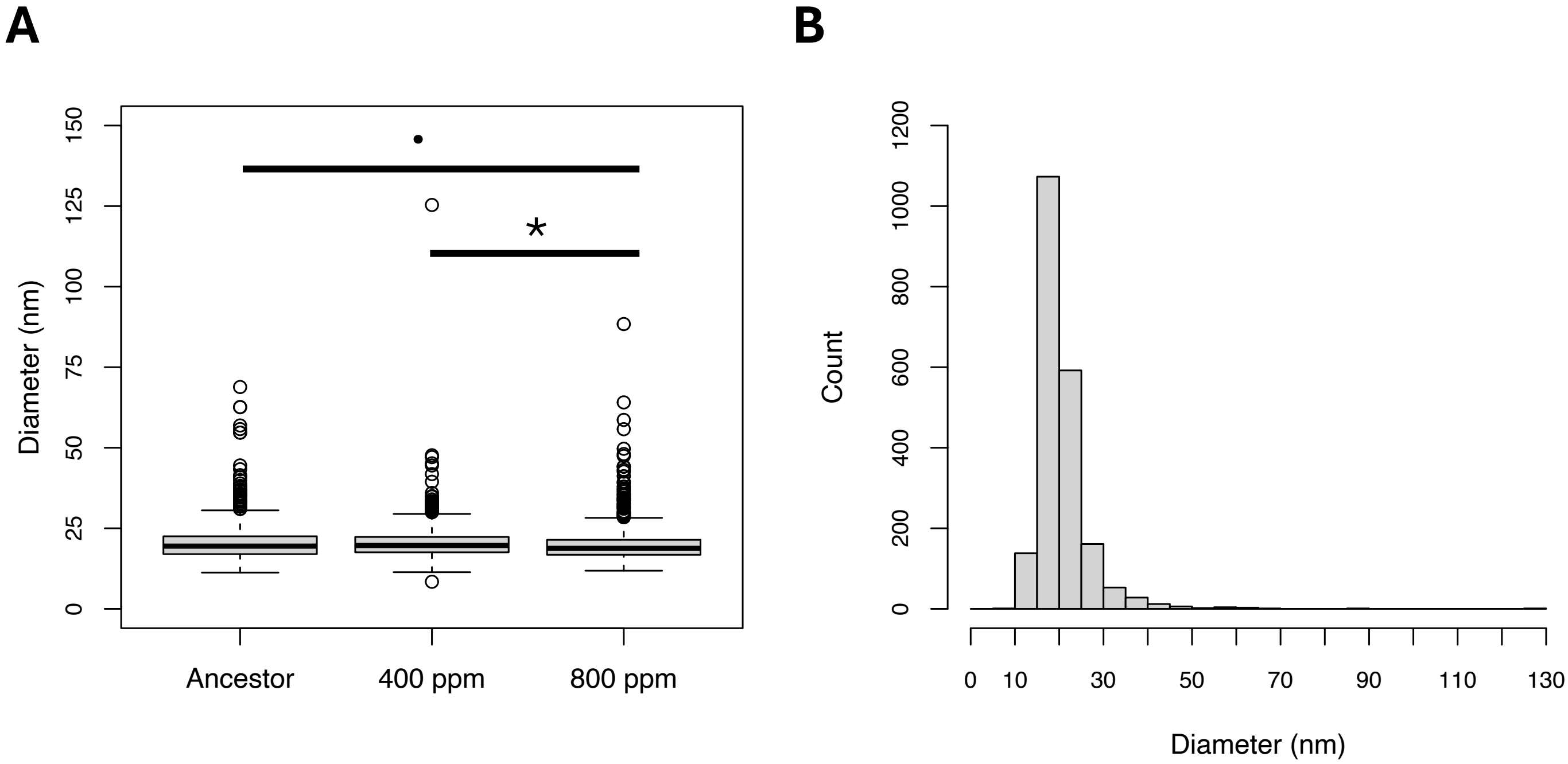

Supplemental Figure 16. Size distribution of *Alteromonas* EVs as estimated by cryoEM. EVs were visualized by cryoEM and measured using ImageJ. **(A)** Whiskers extend to 1.5 times the interquartile range, with outlying values plotted as circles. Significance values are based on extended marginal means analysis of a linear model after logarithmic transformation of EV sizes: *, $p < 0.05$; •, $p < 0.1$. **(B)** Histogram of EV diameters showing the strong right skew.

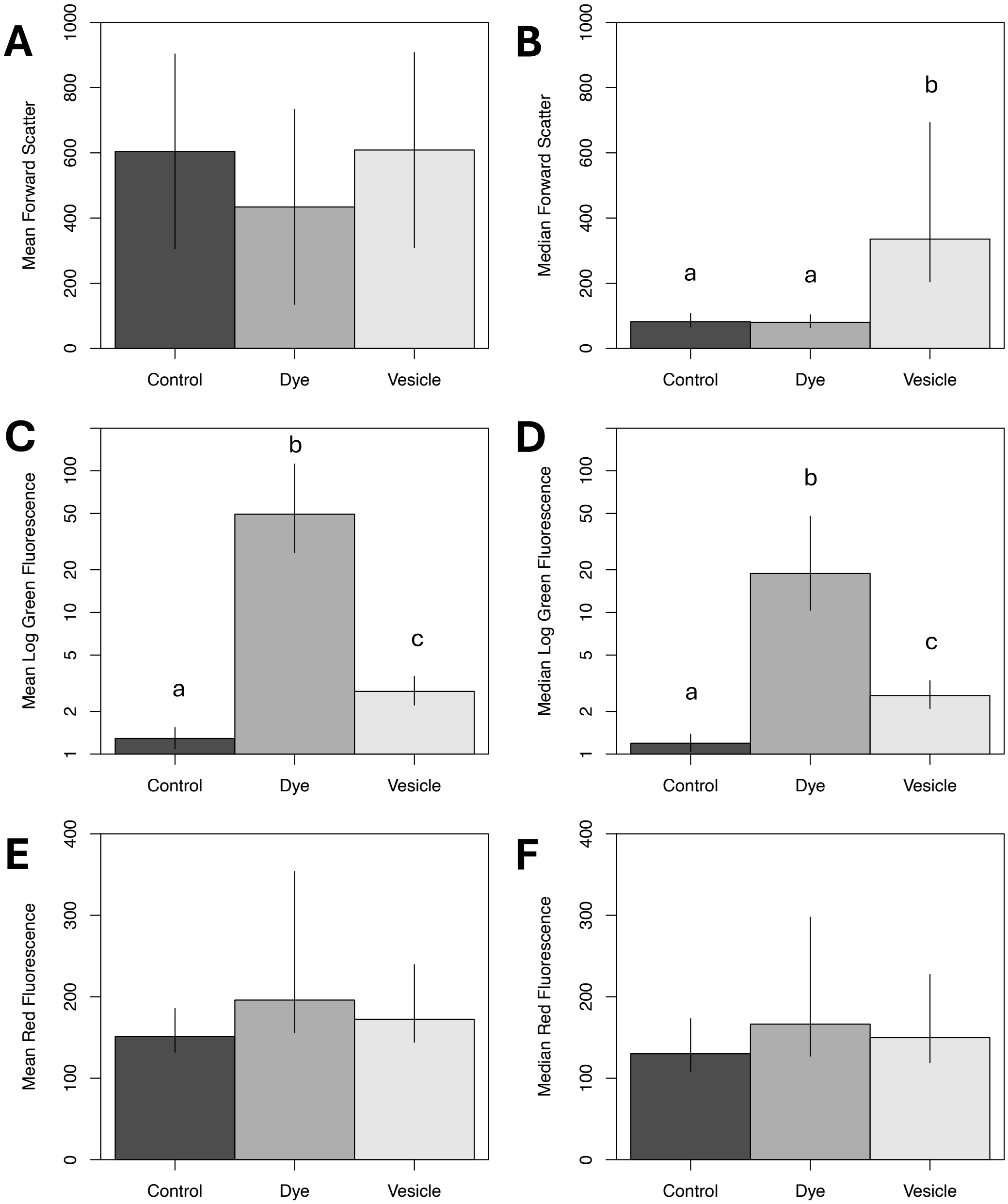

Supplemental Figure 17. Impact of vesicles on flow cytometry signature of *Prochlorococcus*. *Prochlorococcus* cells were observed by flow cytometry either unmodified (control), after staining with Alexa Fluor 488 5-SDB ester dye (Dye), or after being mixed with similarly stained Alteromonas >50kD concentrates (Vesicle). Error bars indicate the 95% confidence intervals from linear model estimates. Letters above bars indicate groups that are significantly different from each other based on those linear models ($p < 0.001$); where no letters are present, there were no differences between treatment groups. Values are derived from events in the *Prochlorococcus* gate, i.e., the upper right quadrant shown in Fig. S1A. **(A)** Mean and **(B)** median forward scatter (size); **(C)** Mean and **(D)** median log green (dye) fluorescence; **(E)** Mean and **(F)** median red (chlorophyll) fluorescence

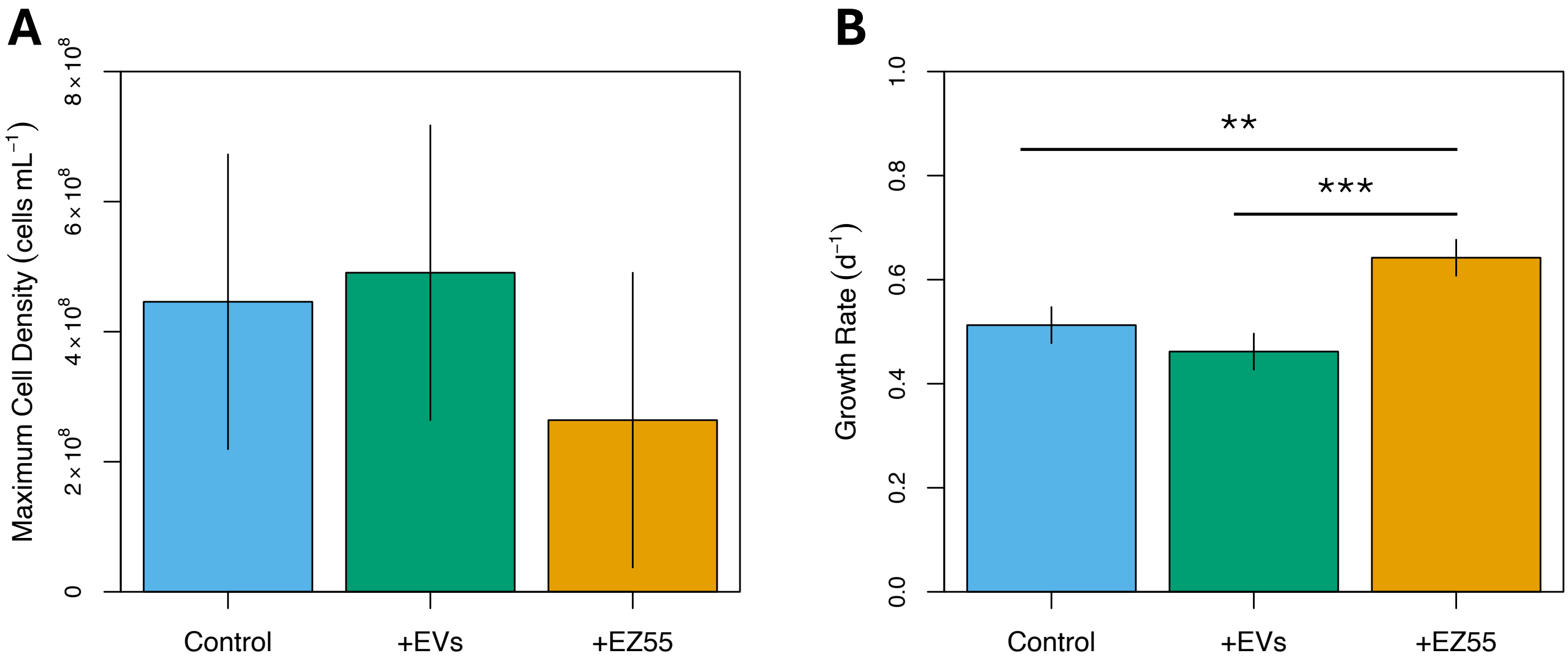

Supplemental Figure 18. Influence of purified *Alteromonas* EVs on *Prochlorococcus* growth. *Prochlorococcus* was grown either axenically (Control) or with the addition of purified *Alteromonas* EVs or live *Alteromonas* EZ55 cells. **(A)** Maximum observed cell density. **(B)** Exponential growth rate. Error bars represent the 95% confidence interval of the estimated mean of three replicates from a linear model. Asterisks indicate the result of post-hoc pairwise extended marginal means comparisons: **, P <0.01; ***, P < 0.001.

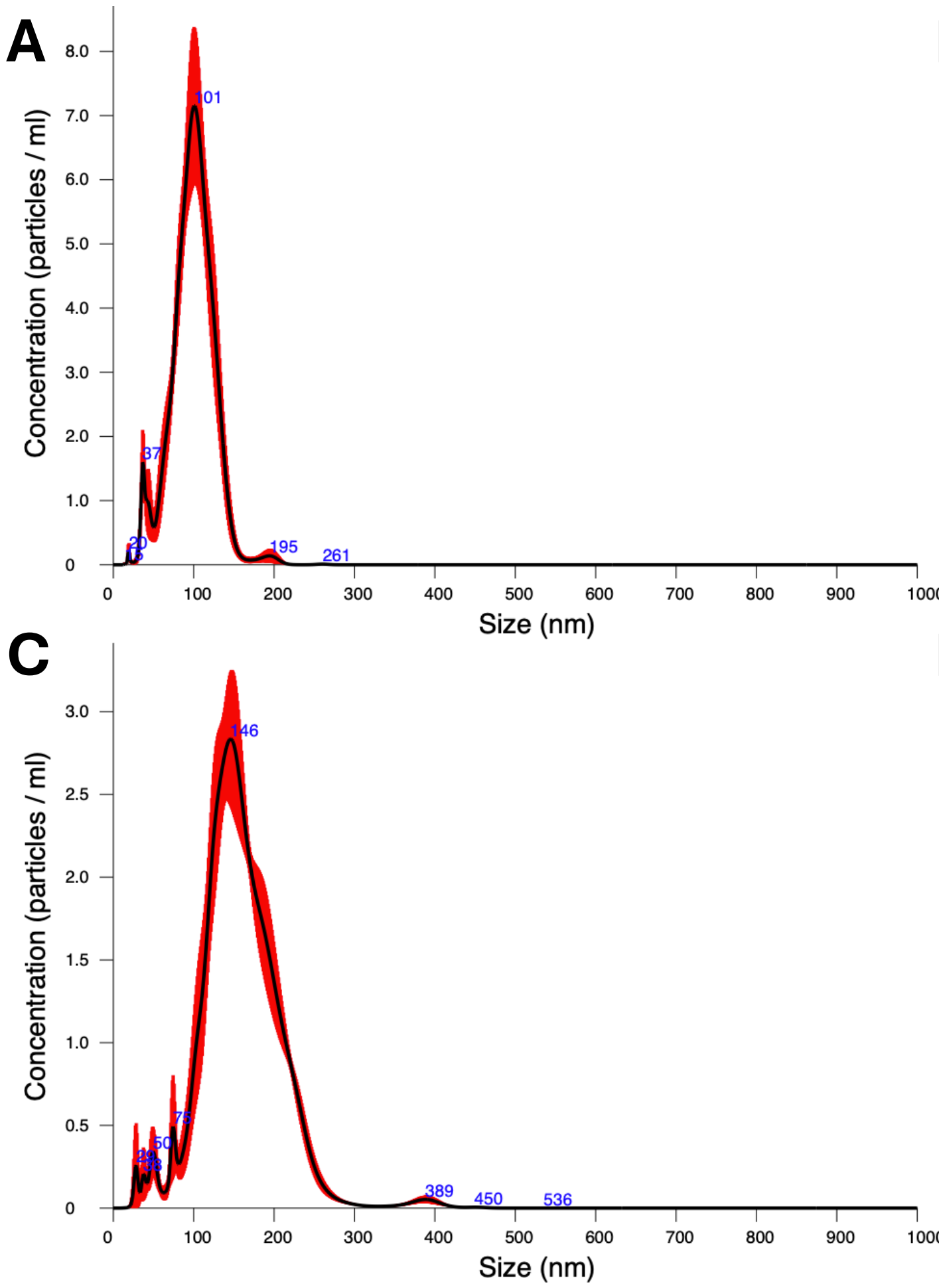

Supplemental Figure 19. Size distributions of *Alteromonas* EVs as determined by nanoparticle imaging. Purified EVs were examined using a Nanosight nanoparticle analzyer. Curves were averaged across five replicate aliquots; the black line represents the mean and the red zone represents +/- 1 standard error. **(A)** Purified *Alteromonas* EVs used to initiate the experiments depicted in Fig. 6. **(B)** EVs purified from axenic *Prochlorococcus* cultures at the end of the experiment (Day 39). **(C)** EVs purified from *Prochlorococcus* cultures treated with live *Alteromonas* cells at the end of the experiment. **(D)** EVs purified from *Prochlorococcus* cultures initially treated with the *Alteromonas* EVs shown in Panel A. The means in panels B, C, and D were greater than A, but size distributions were not significantly different based on pairwise t-tests using the mean and standard deviation output by the Nanosight software ($P > 0.05$).

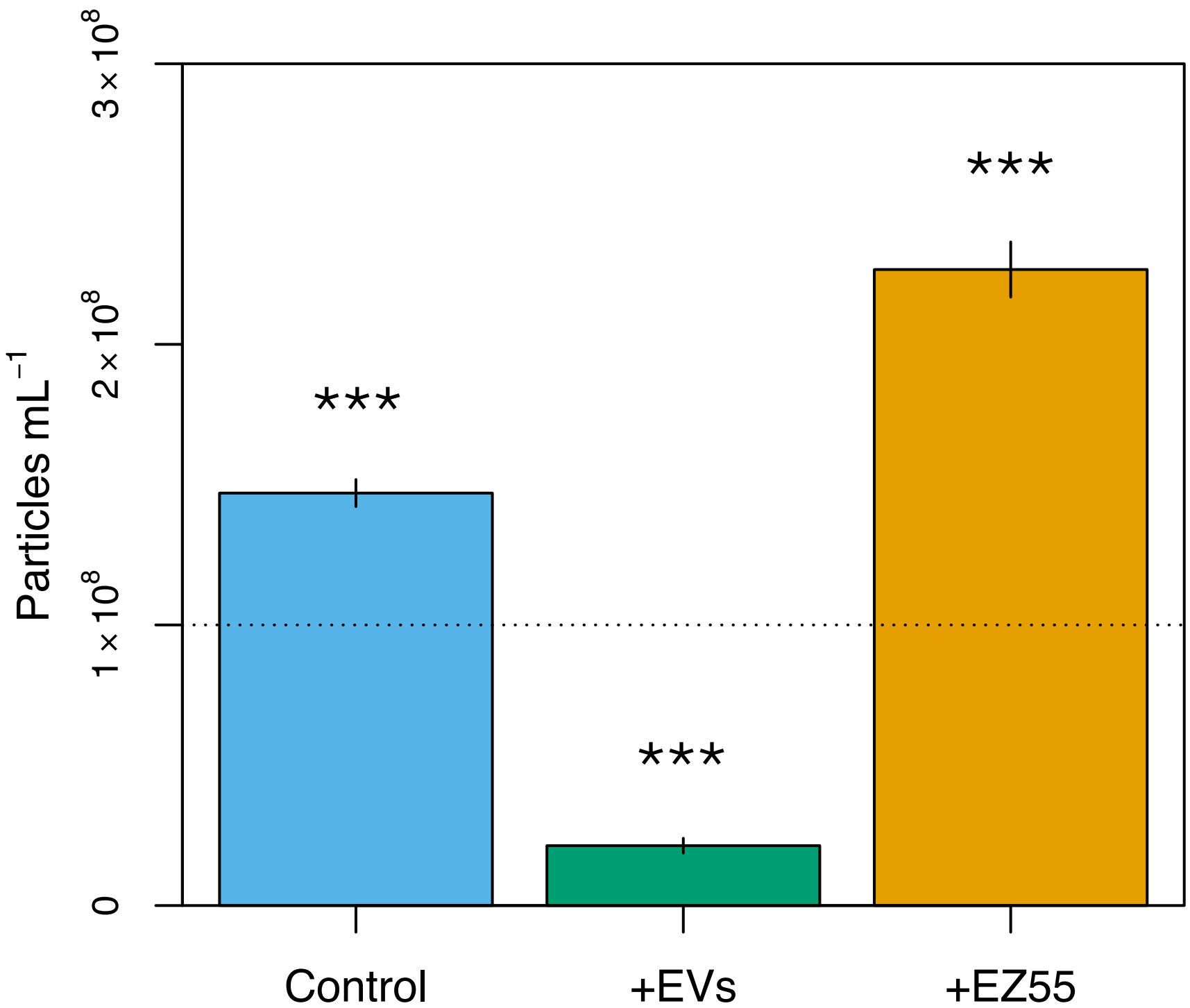

Supplemental Figure 20. Concentration of EVs at the end of the EV addition experiment. Purified EVs were examined using a Nanosight nanoparticle analzyer. Bars represent the mean particle concentration from 5 replicate aliquots, and error bars are the standard deviation. Dashed horizontal line indicates the inoculum concentration of EVs at the beginning of the experiment. Asterisks indicate the result of pairwise one-sample t-test comparisons vs. the inoculum value: ***, $P < 0.001$

Table S1. Peptides identified in culture supernatants or lysates of *Alteromonas*.

Provided as a .csv file with the supplemental material.
